# DNA-PKcs Phosphorylates Cofilin2 to Induce Endothelial Dysfunction and Microcirculatory Disorder in Endotoxemic Cardiomyopathy

**DOI:** 10.34133/research.0331

**Published:** 2024-03-26

**Authors:** Yingzhen Du, Pingjun Zhu, Yukun Li, Jiachi Yu, Tian Xia, Xing Chang, Hang Zhu, Ruibing Li, Qingyong He

**Affiliations:** ^1^ The Second Medical Center & National Clinical Research Center for Geriatric Diseases, Chinese PLA General Hospital, Medical School of Chinese PLA, Beijing 100853, China.; ^2^ Department of Cardiology, Beijing Anzhen Hospital, Capital Medical University, Beijing 100029, China.; ^3^ The First Medical Centre, Medical School of Chinese People’s Liberation Army, Beijing, China.; ^4^ Guang’anmen Hospital, China Academy of Chinese Medical Sciences, Beijing 100053, China.

## Abstract

The presence of endotoxemia is strongly linked to the development of endothelial dysfunction and disruption of myocardial microvascular reactivity. These factors play a crucial role in the progression of endotoxemic cardiomyopathy. Sepsis-related multiorgan damage involves the participation of the catalytic subunit of DNA-dependent protein kinase (DNA-PKcs). However, whether DNA-PKcs contributes to endothelial dysfunction and myocardial microvascular dysfunction during endotoxemia remains unclear. Hence, we conducted experiments in mice subjected to lipopolysaccharide (LPS)-induced endotoxemic cardiomyopathy, as well as assays in primary mouse cardiac microvascular endothelial cells. Results showed that endothelial-cell-specific *DNA-PKcs* ablation markedly attenuated DNA damage, sustained microvessel perfusion, improved endothelial barrier function, inhibited capillary inflammation, restored endothelium-dependent vasodilation, and improved heart function under endotoxemic conditions. Furthermore, we show that upon LPS stress, DNA-PKcs recognizes a TQ motif in cofilin2 and consequently induces its phosphorylation at Thr^25^. Phosphorylated cofilin2 shows increased affinity for F-actin and promotes F-actin depolymerization, resulting into disruption of the endothelial barrier integrity, microvascular inflammation, and defective eNOS-dependent vasodilation. Accordingly, cofilin2-knockin mice expressing a phospho-defective (T25A) cofilin2 mutant protein showed improved endothelial integrity and myocardial microvascular function upon induction of endotoxemic cardiomyopathy. These findings highlight a novel mechanism whereby DNA-PKcs mediates cofilin2^Thr25^ phosphorylation and subsequent F-actin depolymerization to contribute to endotoxemia-related cardiac microvascular dysfunction.

## Introduction

Endotoxemia refers to the presence of bacterial endotoxins, such as lipopolysaccharide (LPS), in the bloodstream. Endotoxemia causes a spectrum of negative effects on vascular endothelial function, including inflammation, hyperpermeability, and decreased barrier function, conducive to dysregulated hemostasis, hemodynamic disturbances, multiorgan dysfunction, and eventually death [1,2]. Microvascular dysfunction caused by endotoxemia has been directly linked to a reduction in myocardial perfusion, which is recognized as a potential contributor to the progression of endotoxemic cardiomyopathy [3,4]. This progression is driven by secretion of proinflammatory and chemotactic substances, increased vascular permeability, thrombosis, and interruption of myocardial blood flow and oxygen supply, resulting in cardiomyocyte ischemia, myocardial edema, and ultimately heart failure [5–8]. Current treatment for endotoxemic cardiomyopathy is limited to antibiotics and fluid resuscitation, which have been shown to be detrimental to endothelial integrity by altering sheer stress, triggering glycocalyx degradation, and inducing vasoconstriction [9–11]. Hence, it is crucial to gain a comprehensive understanding of the molecular basis underlying endothelial damage and coronary microvascular disorders during endotoxemia in order to develop more efficacious therapeutic approaches.

Microvascular function is critically regulated by endothelial barrier integrity [12], adhesion molecule expression [13], and vascular tone [14]. A well-organized endothelial vascular structure allows balanced solute exchange, which is primarily regulated by cell–cell junction proteins such as vascular endothelial (VE)-cadherin and claudin-5 [15]. Adhesive factors such as intercellular adhesion molecule 1 (ICAM1) and vascular cell adhesion molecule 1 (VCAM1) [16] are scarcely expressed in the intact endothelium but are up-regulated by endotoxemia and facilitate the recruitment of inflammatory cells. Endothelial nitric oxide (NO) synthase (eNOS) is expressed in endothelial cells (ECs) and restricts endothelin-1 (ET-1)-induced vasoconstriction [17], acting as an indispensable compensatory mechanism to maintain luminal diameter and sustain blood flow. After exposure to endotoxemia, barrier resistance is disrupted [18,19], an effect followed by increased vascular permeability and cardiac edema. Increased expression of adhesion molecules represents a maladaptive response to endotoxemia, resulting in the deposition of neutrophils within myocardium with subsequent inflammatory damage of cardiac tissue [20]. As mentioned, the imbalance between ET-1 and NO due to decreased eNOS activity mediates pathological vasoconstriction [21], which not only impairs the availability of nutrients to ECs but also restricts also oxygen diffusion into cardiomyocytes. Therefore, elucidating the upstream regulators of endothelial barrier integrity, adhesion molecule expression, and eNOS activity is paramount to design novel therapeutic options to preserve EC integrity and myocardial microvascular function in the setting of endotoxemia.

Proteins from the cofilin/actin depolymerizing factor (ADF) actin-binding cohort play a pivotal role in orchestrating cytoskeletal dynamics, fostering intercellular dialogues, and overseeing cellular division and movement [22]. Mammals express 3 distinct isoforms within the cofilin family: ADF, cofilin-1, and cofilin-2 [23]. Previous studies described the essential roles of cofilins in modifying endothelial actin dynamics, which is required for angiogenesis and wound healing [24]. Subsequent research underscored the pivotal role of cofilins in upholding endothelial junction integrity through the modulation of actin stress fiber assembly. [25]. In turn, a proinflammatory effect of cofilin activation was described in thrombin-treated ECs [26]. In line with these observations, our prior investigations using a murine model of myocardial microvascular reperfusion injury delineated the direct role of cofilin activation in precipitating endothelial dysfunction [27–29]. Yet, the precise roles of cofilins, along with their regulatory pathways impacting endothelial disruption and myocardial microvascular dysfunction during endotoxemia, remain elusive

The catalytic subunit of the DNA-dependent protein kinase (DNA-PKcs) serves as an essential component of DNA-PK, a nuclear serine/threonine kinase pivotal for DNA repair, and stands as a part of the phosphatidylinositol 3-kinase-related kinase enzymatic family dedicated to DNA restoration. Activation of DNA-PK predominantly arises in response to an array of stressors, encompassing oxidative stress [30], senescence [31], chemotoxic challenges [32], and inflammatory stimuli [33]. Our recent findings, based on a murine model subjected to LPS-triggered acute kidney injury, pinpoint DNA-PKcs as a potent mediator exacerbating tubular cell mortality via the facilitation of mitochondrial fission [34]. Moreover, data from a mouse model of LPS-related multiorgan dysfunction [35] suggested that DNA-PKcs activation plays a role in the development of sepsis-related multiple-organ dysfunction during endotoxemia. Since cofilin2 contains Ser/Thr residues amenable to phosphorylation by DNA-PKCs, we generated gene knockout and knockin mice and conducted in vivo and cell-based assays to test the hypothesis that DNA-PKcs promotes endotoxemia-related endothelial dysfunction and myocardial microvascular disorder by inducing cofilin2-dependent actin cytoskeleton remodeling.

## Results

### Endotoxemia triggers cardiac endothelial DNA damage through DNA-PKcs

**Fig. 1.**
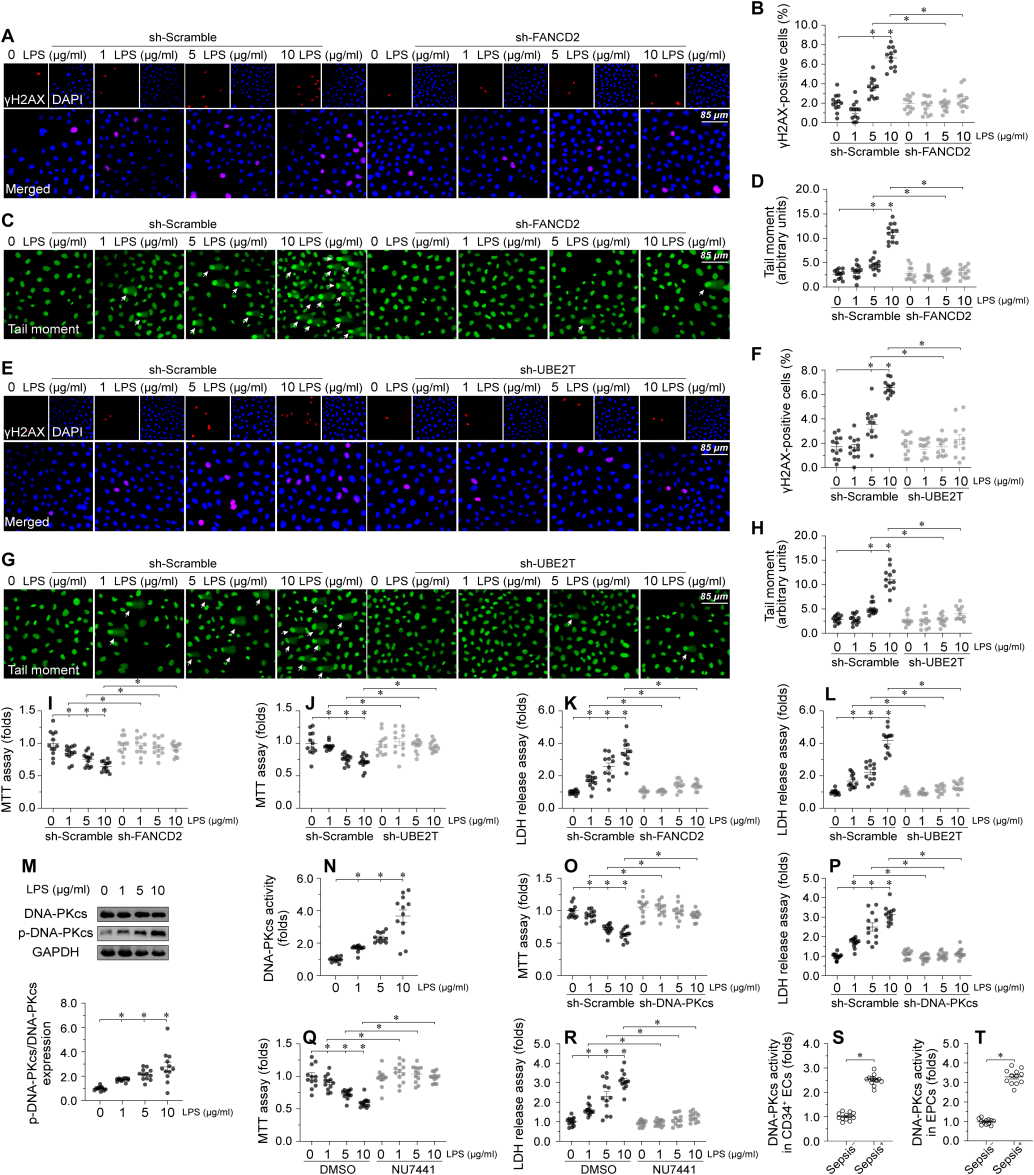
Endotoxemia triggers cardiac endothelial DNA damage through DNA-PKcs. Primary mouse CMECs were transduced with control shRNA (sh-scramble), FANCD2 shRNA (sh-FANCD2), or UBE2T shRNA (sh-UBE2T) before exposure to LPS (0, 1, 5, and 10 μg/ml) for 24 h. (A and B) Representative immunostaining and statistical data for γH2AX-positive CMECs transduced with sh-scramble or sh-FANCD2. (C and D) DNA damage was assessed by comet assays in CMECs transduced with sh-scramble or sh-FANCD2. The arrows indicate nuclei with DNA damage. (E and F) Representative immunostaining and statistical data for γH2AX-positive CMECs transduced with sh-scramble or sh-UBE2T. (G and H) DNA damage was assessed by comet assays in CMECs transduced with sh-scramble or sh-UBE2T. The arrows indicate nuclei with DNA damage. (I and J) MTT assay was used to evaluate cell viability in CMECs infected with sh-FANCD2 or sh-UBE2T. (K and L) ELISA was used to measure LDH levels in culture media from CMECs infected with sh-FANCD2 or sh-UBE2T. (M) Representative western blots and statistical data showing changes in phos-DNA-PKcs expression in LPS-exposed CMECs. (N) ELISA was used to evaluate DNA-PKcs activity in CMECs treated with LPS. (O) MTT assay was used to evaluate cell viability in CMECs transduced with sh-DNA-PKcs or sh-scramble. (P) ELISA was used to measure LDH levels in culture media from CMECs transduced with sh-DNA-PKcs or sh-scramble. (Q) MTT assay was used to evaluate cell viability in CMECs treated with the DNA-PKcs inhibitor NU7441. (R) ELISA was used to measure LDH levels in culture media from CMECs treated with NU7441. (S and T) ELISA was used to evaluate DNA-PKcs activity in human circulating CD34^+^ ECs and EPCs. Experiments were repeated at least 3 times. Data are shown as mean ± SEM (*n* = 6 mice or 3 independent cell isolations per group). *P < 0.05.

To elucidate the DNA damage response of cardiac ECs in the context of endotoxemic challenges, we subjected primary mouse cardiac microvascular ECs (CMECs) to LPS treatment in vitro. LPS elicited dose-dependent DNA damage, evidenced by increased expression of DNA double-strand break markers (i.e. γH2AX; Fig. 1A and B and Fig. 1E and F) and results of comet assays (Fig. 1C and D and Fig. 1G and H). To confirm the causal relationship between DNA damage and EC death, short hairpin RNAs (shRNAs) were used to knockout FANCD2 and UBE2T, 2 necessary components of the DNA damage response. Knockout of either FANCD2 (Fig. 1A to D) or UBE2T (Fig. 1E to H) markedly reduced DNA damage and increased EC viability, as assessed by 3-(4,5-dimethylthiazol-2-yl)-2,5-diphenyltetrazolium Bromide (MTT) (Fig. 1I and J) and lactate dehydrogenase (LDH) release (Fig. 1K and L) experiments. By comparison, overexpression of UBE2T or FANCD2 dose-dependently stimulated the DNA damage response (Fig. S1A to D) and induced EC death (Fig. S1E to H) under both physiological conditions and upon LPS exposure. The above findings suggested that endotoxemia-mediated DNA damage triggers cardiac EC death.

Since DNA breaks are recognized by the Ku70/Ku80 heterodimer (the second, activating subunit of DNA-PK), we next investigated the involvement of both DNA-PKcs and Ku80 on the endotoxemia-related DNA damage response in cardiac ECs. Western blots demonstrated that phos-DNA-PKcs (Fig. 1M) and Ku80 (Fig. S1I and J) protein levels were dose-dependently up-regulated by LPS. In addition, enzyme-linked immunosorbent assay (ELISA) results showed that DNA-PKcs activity (Fig. 1N) as well as Ku80 activity (Fig. S1K) were dose-dependently elevated in CMECs exposed to LPS. Using Adenovirus shRNA vectors and MTT and LDH release assays, we found that silencing DNA-PKcs (Fig. 1O and P) but not Ku80 (Fig. S1L and M) markedly prevented LPS-mediated EC damage. In turn, incubation with NU7441, a specific inhibitor of DNA-PKcs, moderately improved cell viability in the presence of LPS stress (Fig. 1Q and R), whereas treatment with the Ku80 inhibitor STL127705 was without effect (Fig. S1N and O). A dose-dependent increase in DNA-PKcs expression, in contrast to Ku80, results in a reduction of EC viability under both physiological conditions and upon LPS exposure (Fig. S1P to S).

In our further analysis, circulating CD34^+^ ECs—known to play roles in angiogenesis and myocardial microcirculation repair during endotoxemia—as well as endothelial progenitor cells (EPCs) from the peripheral blood of septic patients, exhibited heightened DNA-PKcs activity (Fig. 1S and T) relative to healthy counterparts. Notably, in these septic individuals, elevated DNA-PKcs activity in circulating CD34^+^ ECs correlated with amplified Acute Physiology and Chronic Health Evaluation (APACHE) II and Sequential Organ Failure Assessment (SOFA) scores, diminished left ventricular ejection fraction (LVEF), and escalated lactic acid concentrations (Table S1).

### Deletion of DNA-PKcs sustains myocardial microvascular perfusion during endotoxemic stress

**Fig. 2.**
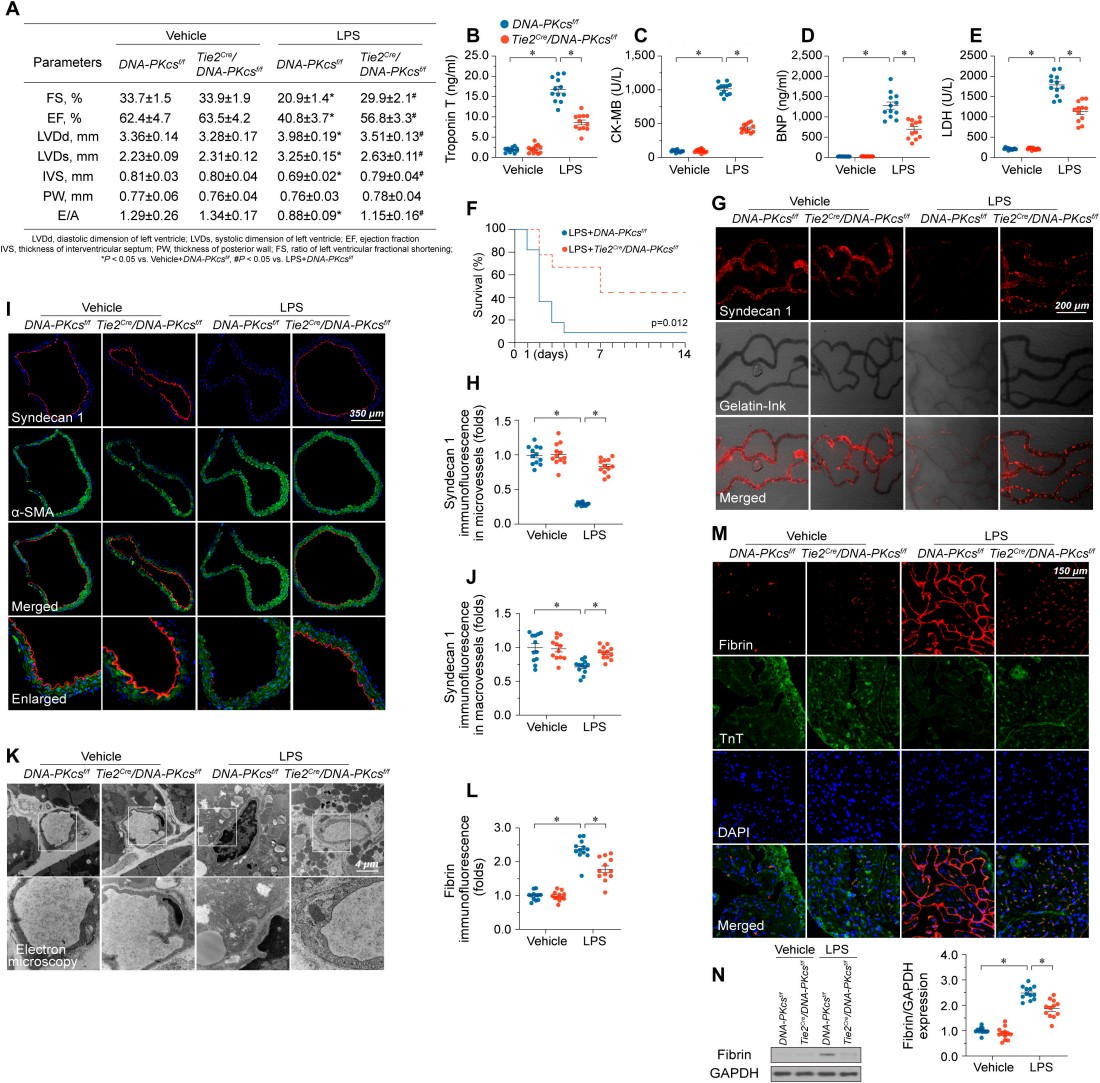
DNA-PKcs deletion attenuates endotoxemia-mediated myocardial microvascular dysfunction. (A) *DNA-PKcs^f/f^* mice were bred to *Tie2^Cre^* mice to generate endothelial-cell-specific *DNA-PKcs* knockout (*DNA-PKcs^f/f^*/*Tie2^Cre^*) mice. Endotoxemic cardiomyopathy was induced via LPS (10 mg/kg) injection and heart function was assessed by echocardiography 48 h later. (B to E) Analysis of serum TnT, CK-MB, BNP, and LDH levels in *DNA-PKcs^f/f^*/*Tie2^Cre^* and control *DNA-PKcs^f/f^* mice. (F) Survival data for *DNA-PKcs^f/f^*/*Tie2^Cre^* and control *DNA-PKcs^f/f^* mice in the presence of LPS. (G and H) Cardiac microvascular imaging after gelatin-ink perfusion and syndecan-1 immunofluorescence in heart tissue from *DNA-PKcs^f/f^*/*Tie2^Cre^* and control *DNA-PKcs^f/f^* mice. (I and J) Syndecan-1 immunofluorescence in aortae isolated from *DNA-PKcs^f/f^*/*Tie2^Cre^* and control *DNA-PKcs^f/f^* mice. α-SMA was used to stain smooth muscle. (K) TEM analysis of ultrastructural alterations in microvessels from *DNA-PKcs^f/f^*/*Tie2^Cre^* and control *DNA-PKcs^f/f^* mice. (L and M) Fibrin immunofluorescence in myocardial microvessels from *DNA-PKcs^f/f^*/*Tie2^Cre^* and control *DNA-PKcs^f/f^* mice. (N) Western blot analysis of fibrin expression in cardiac microvessels from *DNA-PKcs^f/f^*/*Tie2^Cre^* and control *DNA-PKcs^f/f^* mice. Experiments were repeated at least 3 times. Data are shown as mean ± SEM (*n* = 6 mice or 3 independent samples per group). *P < 0.05.

To elucidate the pathological function of DNA-PKcs in endotoxemia, we generated EC-specific DNA-PKcs knockout mice by crossbreeding *Tie2^Cre^* mice with *DNA-PKcs^f/f^* mice. To induce endotoxemic cardiomyopathy, the animals received an intraperitoneal injection of LPS for 72 h. *Tie2^Cre^* mice cross-bred with *Ku80^f/f^* mice were also generated to assess whether endotoxemia-related cardiac microvascular dysfunction is exclusively associated with DNA-PKcs. The knockdown efficiency was confirmed by western blots (Fig. S1A and B). LPS treatment markedly disrupted myocardial function, as evidenced by mildly decreased relaxation function and a slight reduction in contraction parameters (Fig. 2A and Fig. S2C). Besides, the serum levels of myocardial injury biomarkers, i.e. trinitrotoluene (TNT), creatine kinase MB (CK-MB), B‐type natriuretic peptide (BNP), and LDH, exhibited a significant increase (Fig. 2B to E and Fig. S2D to G), while the survival rate decreased by approximately 10% in LPS-treated *DNA-PKcs^f/f^* mice (Fig. 2F) compared to LPS-treated *Tie2^Cre^*/*DNA-PKcs^f/f^* mice. However, no difference of survival rate was observed between *Tie2^Cre^*/*Ku80^f/f^* mice and LPS-treated *Ku80^f/f^* mice (Fig. S2H). Gelatin-ink perfusion experiments were next used to assess microvascular patency, while transmission electron microscopy (TEM) was applied to detect ultrastructural changes in cardiac microvessels. LPS exposure induced microcirculatory occlusion in *DNA-PKcs^f/f^* mice, an effect accompanied by reduced expression of syndecan-1, a core component of the endothelial glycocalyx (Fig. 2G and H). Similar alterations were also noted in aortae isolated from *DNA-PKcs^f/f^* mice (Fig. 2I and J). TEM analysis showed that LPS caused endothelial swelling, microvascular wall destruction, and luminal stenosis (Fig. 2K). Interestingly, deletion of *DNA-PKcs* (Fig. 2K) but not *Ku80* (Fig. S2I) in ECs not only preserved endothelial ultrastructure but also improved coronary microvascular perfusion. Accordingly, fibrin immunofluorescence confirmed that intravascular coagulation was reduced in myocardial microvessels from LPS-treated *Tie2^Cre^*/*DNA-PKcs^f/f^* mice (Fig. 2L and M). Western blot analysis further detected the deposition of fibrin in capillaries from LPS-treated *DNA-PKcs^f/f^* mice and *Ku80^f/f^* mice, and this effect was reduced in *Tie2^Cre^/DNA-PKcs^f/f^* mice (Fig. 2N) but not in *Tie2^Cre^*/*Ku80^f/f^* mice (Fig. S2J).

To prevent the activation of DNA-PKcs without influencing its expression, NU7441, a specific inhibitor of DNA-PKcs, was administered to wild-type (WT) mice before LPS injection. Similar to the results observed in *Tie2^Cre^*/DNA-PKcs*^f/f^* mice, pharmacological in vivo inhibition of DNA-PKcs largely reversed myocardial contraction/relaxation dysfunction (Fig. S3A) and markedly prevented the up-regulation of TnT, CK-MB, BNP, and LDH in LPS-treated WT mice (Fig. S3B to E). Consistent with these findings, NU7441 treatment increased mouse survival rate (Fig. S3F) and abolished LPS-mediated microvessel occlusion and ultrastructural abnormalities in in situ cardiac ECs (Fig. S3G). Moreover, LPS failed to induce fibrin accumulation within capillaries (Fig. S3H) in NU7441-treated WT mice. These data indicated that DNA-PKcs inactivation ameliorates endotoxemia-mediated myocardial microvascular injury. In light of the observed impact of DNA-PKcs pharmacological inhibition on cardiomyocytes during LPS exposure (Fig. S3I and J), our further investigations utilized *Tie2^Cre^/DNA-PKcs^f/f^* mice. This approach was specifically chosen to explore the role of DNA-PKcs in modulating the cardiac microvasculature.

### DNA-PKcs contributes to endotoxemia-induced barrier dysfunction, cardiac inflammation, and myocardial vasoconstriction

**Fig. 3.**
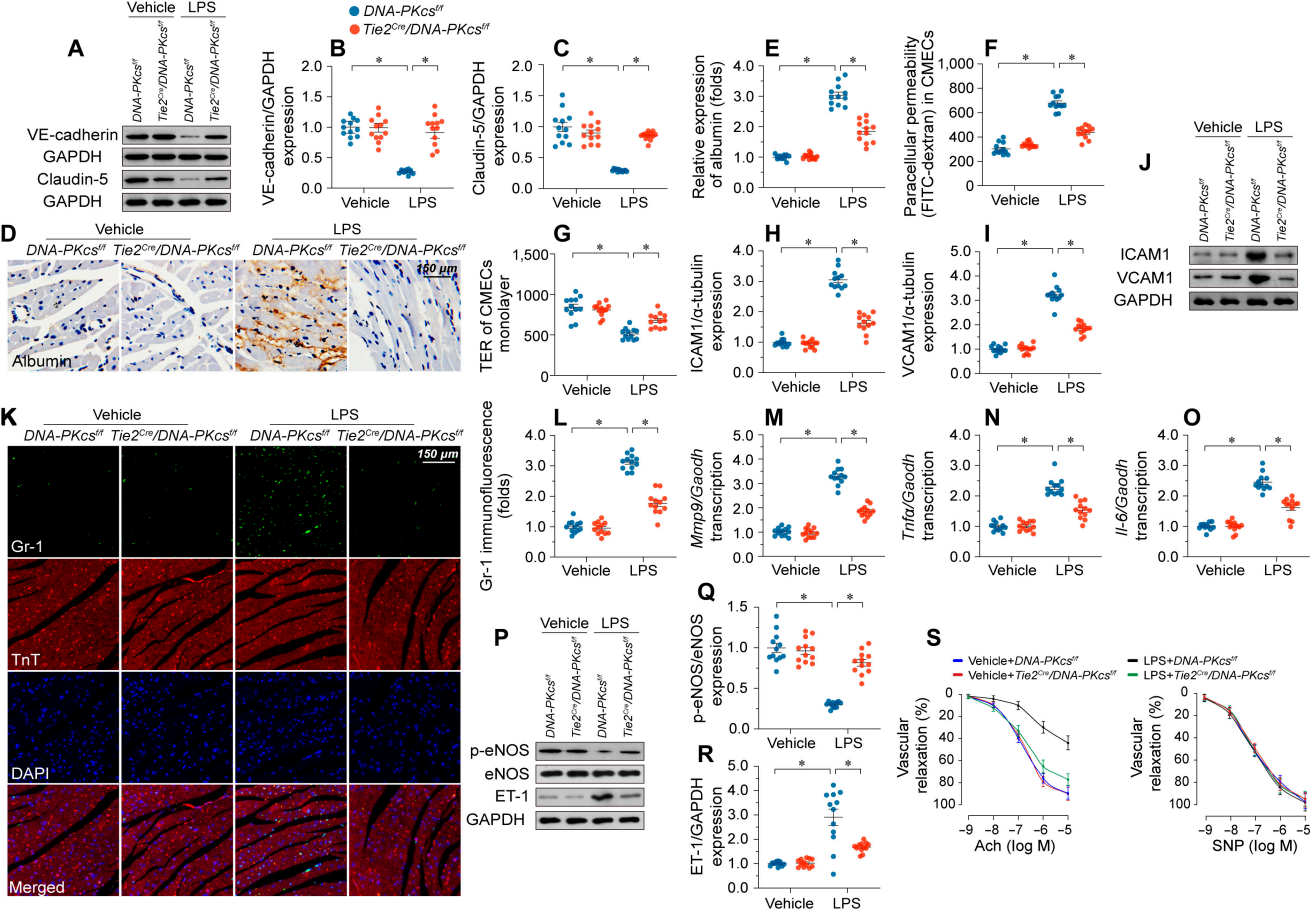
DNA-PKcs promotes endothelial barrier dysfunction, inflammation, and vasoconstriction in myocardial microvessels. (A to C) Western blot analysis of claudin-5 and VE-cadherin expression in *DNA-PKcs^f/f^*/*Tie2^Cre^* and control *DNA-PKcs^f/f^* mice. (D and E) Immunohistochemistry was used to observe albumin leakage into myocardium in *DNA-PKcs^f/f^*/*Tie2^Cre^* and control *DNA-PKcs^f/f^* mice. (F) FITC-dextran clearance assays were performed following 24-h LPS treatment (10 μg/ml) in CMECs isolated from *DNA-PKcs^f/f^*/*Tie2^Cre^* and control *DNA-PKcs^f/f^* mice. FITC-dextran permeation was measured to assess alterations in endothelial barrier function. (G) TER assays were performed in CMECs isolated from *DNA-PKcs^f/f^*/*Tie2^Cre^* and control *DNA-PKcs^f/f^* mice to evaluate changes in endothelial barrier integrity following LPS exposure. (H to J) Western blot analysis of VCAM1 and VCAM1 expression in cardiac microvessels from *DNA-PKcs^f/f^*/*Tie2^Cre^* and control *DNA-PKcs^f/f^* mice treated with LPS. (K and L) Immunofluorescence of Gr-1^+^ neutrophils in heart samples from *DNA-PKcs^f/f^*/*Tie2^Cre^* and control *DNA-PKcs^f/f^* mice treated with LPS. DAPI was used to stain nuclei and TnT was used to stain cardiomyocytes. (M to O). RT-qPCR was used to analyze the transcription of *Mmp-9*, *IL-6*, and *Tnf-α* in cardiac tissue from *DNA-PKcs^f/f^*/*Tie2^Cre^* and control *DNA-PKcs^f/f^* mice after LPS exposure. (P to R) Western blot analysis of p-eNOS and ET-1 expression in heart tissues from *DNA-PKcs^f/f^*/*Tie2^Cre^* and control *DNA-PKcs^f/f^* mice treated with LPS. (S) Endothelial-dependent and endothelial-independent relaxation responses were assessed in aortic rings by applying Ach (10^−9^–10^−5^ M) or SNP (10^−10^–10^−6^ M). Experiments were repeated at least 3 times. Data are shown as mean ± SEM (*n* = 6 mice or 3 independent cell isolations per group). *P < 0.05.

Since endothelial integrity and microvascular function are critically dependent on endothelial barrier integrity, cell adhesion protein expression, and vascular tone, we subsequently investigated the impact of DNA-PKcs inactivation on these variables. After exposure to LPS, the expression of barrier-related proteins VE-cadherin and claudin-5 (Fig. 3A to C) was markedly down-regulated in heart tissues, an alteration that contributed to the permeation of albumin into myocardium (Fig. 3D and E). Genetic deletion of endothelial *DNA-PKcs*, but not *Ku80* was able to reverse the down-regulation of VE-cadherin and claudin-5 (Fig. 3A to C and Fig. S4A to C) and prevented albumin leakage (Fig. 3D and E and Fig. S4D and E). To validate these findings in an in vitro setting, we performed fluorescein isothiocyanate-dextran (FITC-dextran) clearance and transendothelial electrical resistance (TER) assays using cultured CMECs isolated from *DNA-PKcs^f/f^*, *Tie2^Cre^*/*DNA-PKcs^f/f^*, *Ku80^f/f^*, or *Tie2^Cre^*/*Ku80^f/f^* mice. These cells were then treated with LPS in vitro. After LPS exposure, we observed a notable augmentation in FITC-dextran extravasation, indicating increased vascular permeability, and a decrease in TER, indicating compromised endothelial barrier function (Fig. 3F and G). Notably, CMECs derived from *Tie2^Cre^/DNA-PKcs^f/f^* mice (Fig. 3F and G), but not those from *DNA-PKcs^f/f^* mice, or *Tie2^Cre^/Ku80s^f/f^* mice (Fig. S4F and G), exhibited reduced FITC-dextran permeation and restored TER, suggesting that DNA-PKcs deficiency in CMECs plays a pivotal role in maintaining endothelial barrier integrity in response to LPS treatment. Conversely, overexpression of DNA-PKcs, but not Ku80, dose-dependently augmented FITC-dextran passage and reduced TER in CMECs under physiological conditions or LPS treatment (Fig. S4H to K).

Besides impairing EC barrier integrity, LPS increased the expression of cell adhesion molecules, namely ICAM1/VCAM1 (Fig. 3H to J), and promoted both deposition of Gr-1^+^ neutrophils within myocardium (Fig. 3K and L) and transcriptional up-regulation of proinflammatory factors in the heart (Fig. 3M to O). Ablation of *DNA-PKcs*, but not *Ku80*, reduced ICAM1/VCAM1 expression (Fig. 3H to J and Fig. S4L and M), prevented the recruitment of Gr-1^+^ neutrophils in myocardial tissue (Fig. 3K and L and Fig. S4N and O), and attenuated cardiac proinflammatory cytokine transcription (Fig. 3M to O and Fig. S4P to R).

Phosphorylated (activated) eNOS (p-eNOS) mediates NO synthesis in the vascular endothelium. Western blot assays in heart tissues showed that LPS treatment reduced p-eNOS levels and up-regulated ET-1 expression, and these changes were partly abrogated upon deletion of *DNA-PKcs* (Fig. 3P to R) but not of *Ku80* (Fig. S4S to T). Experiments in aortic ring explants further showed that endothelium-dependent vasodilation was blunted by LPS, and this effect was largely reversed after *DNA-PKcs* deletion (Fig. 3S).

Recent studies have reported that deletion of DNA-PKcs reduces the expression of Toll-like receptor (TLR) 1, TLR3, and TLR8 [36]. Furthermore, activation of DNA-PKcs is also associated with a robust immune response [37]. To investigate whether the cardioprotective effect mediated by *DNA-PKcs* deficiency is linked to improved immune reactions, western blots were employed to observe changes in TLR4, NLR family pyrin domain containing 3 (NLRP3), and caspase-1 in heart tissues and CMECs. As depicted in Fig. S5A and B, treatment with LPS up-regulates the levels of TLR4, NLRP3, and caspase-1 in heart tissues, but this change is partially suppressed by *DNA-PKcs* ablation. Similarly, in LPS-treated CMECs, transfection of shRNA against *DNA-PKcs* effectively prevents the up-regulation of TLR4, NLRP3, and caspase-1. These results demonstrate that endothelial-cell-specific knockout of *DNA-PKcs* has the potential to normalize immune reactions in heart tissues.

In accordance with these findings, silencing of *DNA-PKcs*, but not *Ku80*, preserved endothelial barrier function (Fig. S6A to D), reduced ICAM1 expression (Fig. S6E to H), and up-regulated p-eNOS activity (Fig. S6I and J) in LPS-challenged EPCs isolated from the peripheral blood of healthy subjects. These data indicated that DNA-PKcs activation is a key mediator of barrier dysfunction, adhesive molecule expression, and impaired vascular tone in myocardial microvessels exposed to LPS.

### LPS-mediated DNA-PKcs activation promotes cytoskeletal alterations in microvascular ECs

**Fig. 4.**
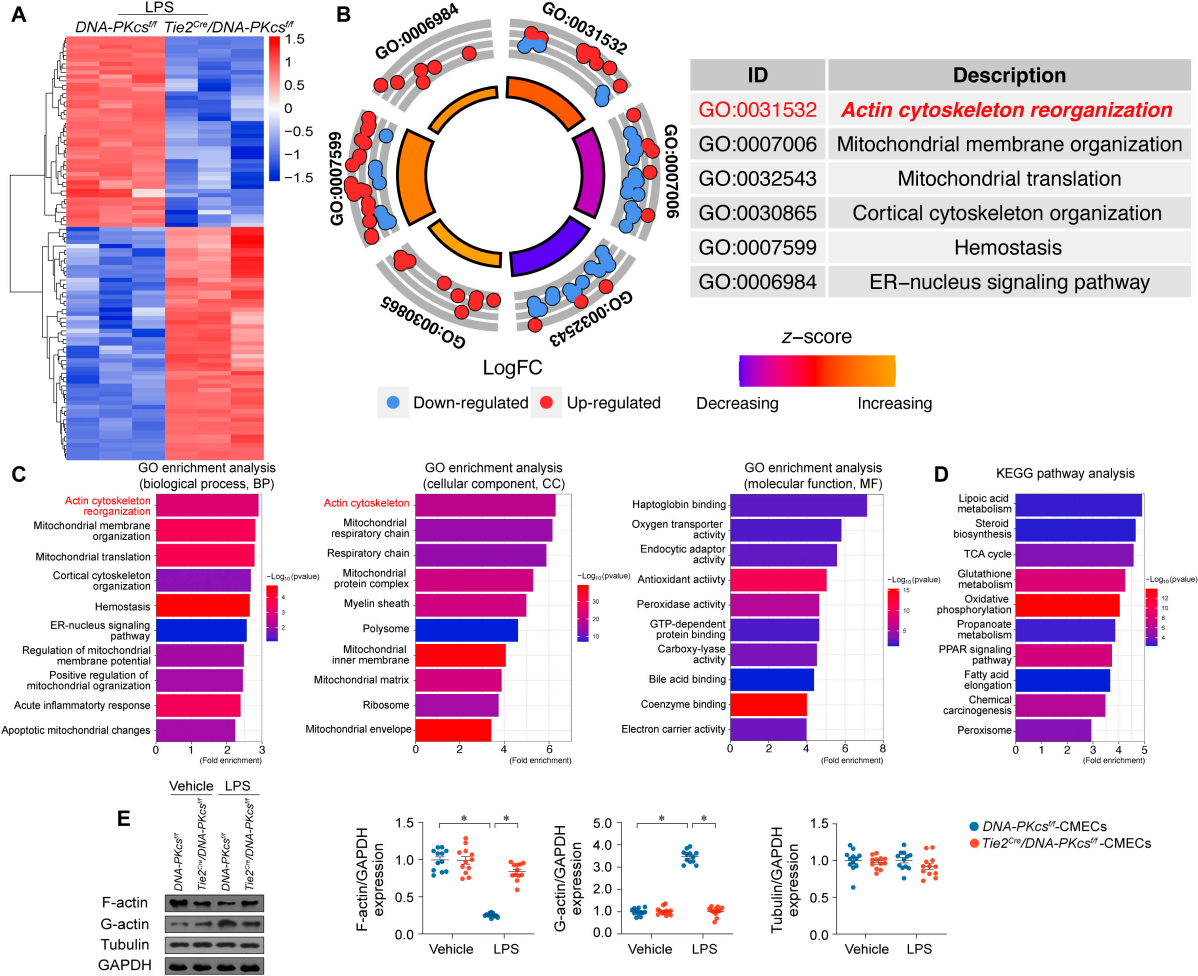
DNA-PKcs depletion regulates the expression of cytoskeleton-related proteins in vascular endothelium. (A) Expression heat map of differentially expressed proteins between CMECs isolated from LPS-treated *DNA-PKcs^f/f^*/*Tie2^Cre^* and control *DNA-PKcs^f/f^* mice in the presence of LPS. Of a total of 1,085 differentially expressed proteins, 490 were up-regulated and 595 were down-regulated by *DNA-PKcs* gene deletion (*n* = 3 independent cell isolations per group). (B and C) GO Cellular Component (CC), GO Molecular Function (MF), and GO Biological Process (BP) enrichment analyses of the 1,085 differentially expressed proteins. (D) Kyoto Encyclopedia of Genes and Genome (KEGG) analysis. (E) Western blot analysis of F-actin and G-actin expression in CMECs isolated from *DNA-PKcs^f/f^*/*Tie2^Cre^* and control *DNA-PKcs^f/f^* mice in the presence of LPS. Experiments were repeated at least 3 times. Data are shown as mean ± SEM (*n* = 3 independent cell isolations per group). *P < 0.05.

To decipher the molecular pathways through which DNA-PKcs deletion mitigates endothelial dysfunction and microvascular damage during endotoxemia, label-free liquid chromatography-mass spectrometry (LC-MS) proteomics was used for unbiased analysis of protein expression profiles in CMECs isolated from *DNA-PKcs^f/f^*/*Tie2^Cre^* or control *DNA-PKcs^f/f^* mice in the presence of LPS. The analysis revealed that *DNA-PKcs* deficiency resulted in altered expression of 1,085 proteins (log_2_FC>1.5), with 490 proteins being up-regulated and 595 proteins being down-regulated (Fig. 4A). Gene Ontology (GO) analysis of the 1,085 proteins showed that actin cytoskeleton reorganization was the most down-regulated pathway in *DNA-PKcs*-deficient cells (Fig. 4B and C). A GO Biological Process query further illustrated that 1,085 proteins were involved in cytoskeleton function (Fig. 4B to D). In sum, proteins governing cytoskeleton homeostasis were primarily regulated by *DNA-PKcs* deficiency in CMECs during LPS exposure. Experimental validation in CMECs provided further confirmation that LPS treatment resulted in the degradation of F-actin into G-actin. Importantly, this effect was reversed in CMECs lacking DNA-PKcs (Fig. 4E). In contrast, the levels of tubulin, a key component of microtubules, remained unaffected by both LPS treatment and *DNA-PKcs* deletion (Fig. 4E). Likewise, in cultured CD34^+^ ECs or EPCs, exposure to LPS resulted in the disassembly of F-actin into G-actin. Notably, this effect was mitigated by the use of shRNA targeting DNA-PKcs (Fig. S7). These data suggested that endotoxemia-related DNA-PKcs activation negatively affects actin homeostasis in CMECs.

### DNA-PKcs promotes endothelial dysfunction by inducing actin cytoskeleton derangement

**Fig. 5.**
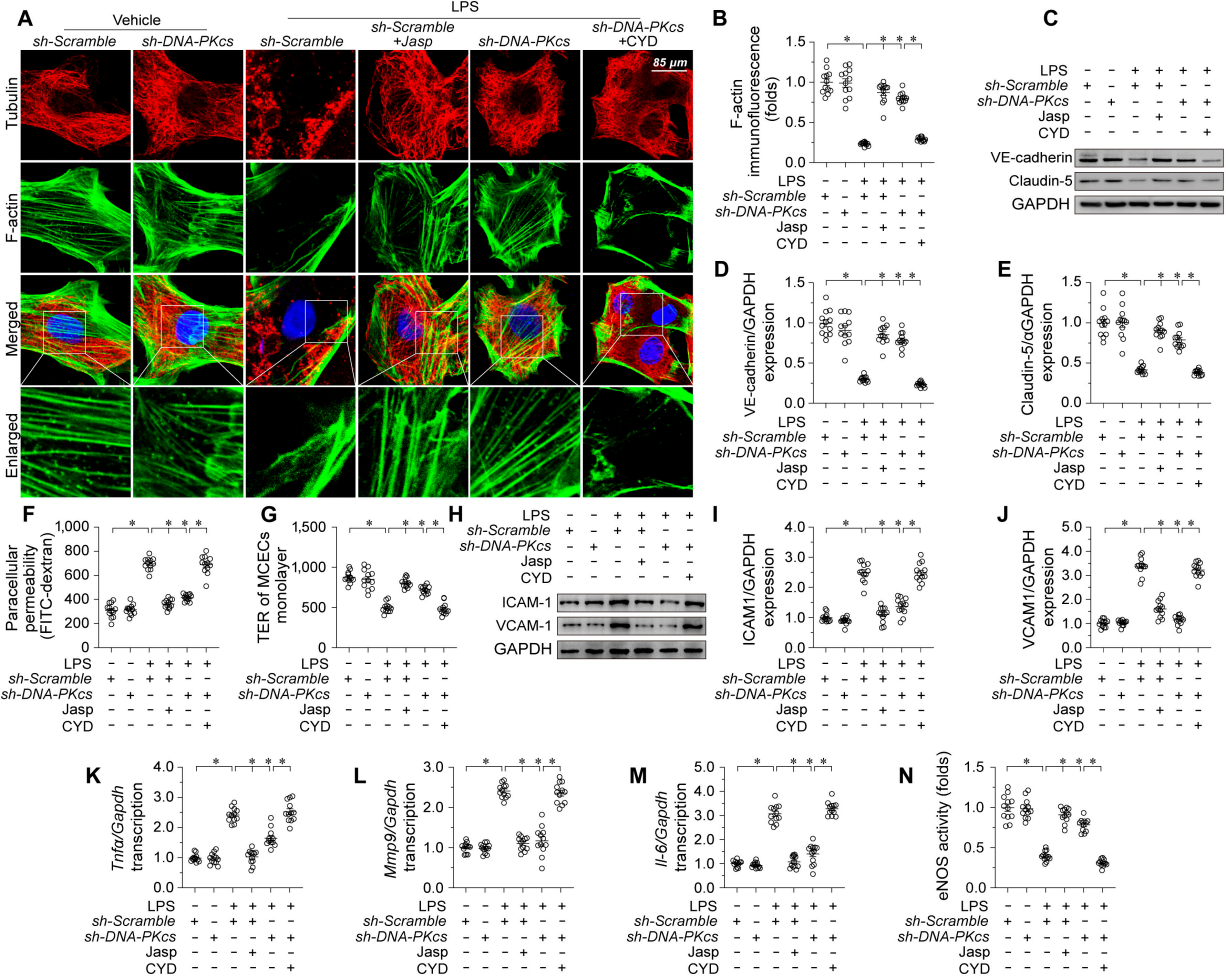
DNA-PKcs mediates endotoxemia-related endothelial dysfunction through disruption of the actin cytoskeleton. CMECs were transduced with sh-DNA-PKcs or sh-Scramble before being exposed to LPS (10 μg/ml) for 24 h. (A and B) F-actin immunofluorescence. Cytochalasin D (CYD) and jasplakinolide were applied to CMECs cultures to induce and prevent, respectively, F-actin depolymerization. Scale bar, 90 μm. (C to E) Western blot analysis of VE-cadherin and claudin-5 expression. (F and G) Evaluation of endothelial barrier function by FITC clearance (F) and TER (G) assays. (H to J) Western blot analysis of ICAM1 and VCAM1 expression. (K to M) RT-qPCR-based analysis of *Il-6*, *Mcp-1,* and *Tnfα* transcription. (N) ELISA-based analysis of eNOS activity. Experiments were repeated at least 3 times. Data are shown as mean ± SEM (*n* = 3 cell isolations per group). *P < 0.05.

To confirm if DNA-PKCs-dependent actin cytoskeleton remodeling is required for LPS-mediated endothelial dysfunction and microvascular damage, actin depolymerization and polymerization were induced in CMECs through exposure to cytochalasin D (CYD) [38] and jasplakinolide [39], respectively. Immunofluorescence assays showed that jasplakinolide treatment attenuated or inhibited LPS-induced F-actin depolymerization (Fig. 5A and B), VE-cadherin/claudin-5 down-regulation (Fig. 5C to E), FITC-dextran permeation and TER reduction (Fig. 5F and G), ICAM1/VCAM1 up-regulation (Fig. 5H to J), proinflammatory cytokine expression (Fig. 5K to M), and eNOS inactivation (Fig. 5N). Confirming a key role for DNA-PKcs in LPS-induced actin cytoskeleton derangement, transfection of sh-DNA-PKcs failed to attenuate or inhibit the above phenomena in CYD-pretreated, LPS-exposed CMECs (Fig. 5A to N). These results confirmed that during endotoxemic stress DNA-PKcs activation induces endothelial dysfunction by promoting F-actin depolymerization and disrupting cytoskeletal stability.

### DNA-PKcs phosphorylates cofilin2 at Thr^25^

**Fig. 6.**
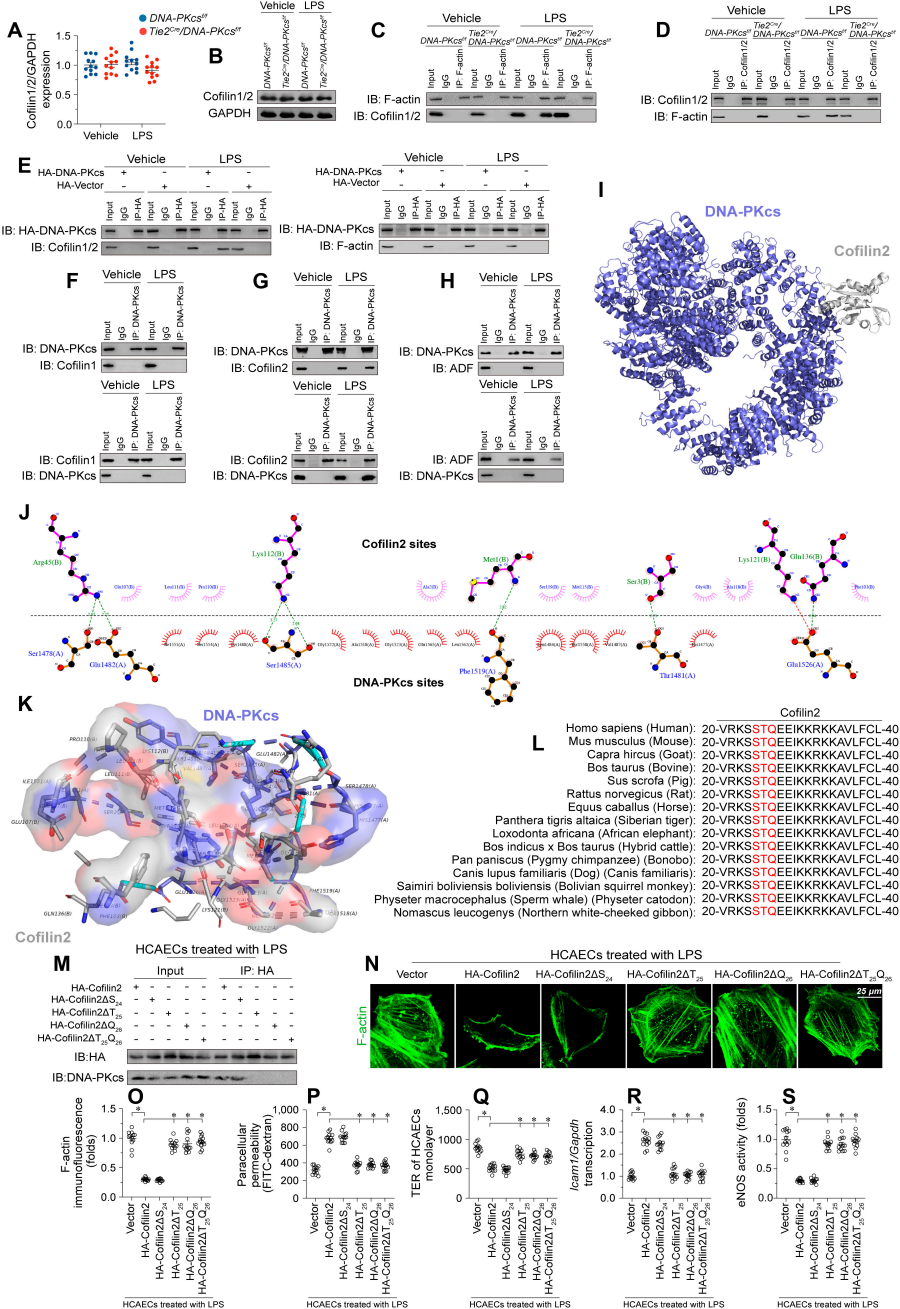
DNA-PKcs binds to cofilin2 by recognizing a TQ motif. (A and B) Western blot analysis of Cofilin1/2 in CMECs isolated from *DNA-PKcs^f/f^*/*Tie2^Cre^* and control *DNA-PKcs^f/f^* mice in the presence of LPS. (C and D) Co-IP assays were conducted to evaluate binding of DNA-PKcs to cofilin1/2 and F-actin using extracts from LPS-treated CMECs isolated from *DNA-PKcs^f/f^*/*Tie2^Cre^* and control *DNA-PKcs^f/f^* mice. (E) HCAECs were transfected with HA-DNA-PKCs before LPS treatment. Then, the interaction between DNA-PKcs and F-actin as well as the interplay between DNA-PKcs and cofilin1/2 were measured through Co-IP. (F) Immunoblots of DNA-PKcs and cofilin1 immunoprecipitates (Ips) in LPS-treated HCAECs. (G) Immunoblots of DNA-PKcs and cofilin2 Ips in LPS-treated HCAECs. (H) DNA-PKcs and ADF Ips from LPS-treated HCAECs were immunoblotted as indicated. (I) Docking analysis of the interaction between DNA-PKcs and cofilin2. (J and K) Putative hydrogen and hydrophobic bonds between DNA-PKcs and cofilin2 are indicated. (L) Amino acid sequences of cofilin2 in various species. (M) HCAECs were transfected with His-tagged cofilin2 constructs, including cofilin2 without Ser^24^ (His-cofilin2ΔS_24_), without Thr^25^ (His-cofilin2ΔT_25_), without Gln^26^ (His-cofilin2ΔQ_26_), or without Thr^25^ and Gln^26^ (His-cofilin2ΔT_25_Q_26_). After cells were exposed to LPS, His immunoprecipitates were collected and immunoblotted to determine their interaction with DNA-PKcs. (N and O) Immunofluorescence analysis of F-actin expression in HCAECs transfected with different mutant cofilin2 constructs. (P and Q) Endothelial barrier function was determined by FITC clearance assay and TER detection in HCAECs transfected with different cofilin2 constructs. (R) qPCR analysis of *Icam1* expression in HCAECs transfected with different cofilin2 constructs. (S) ELISA assay of eNOS activity in HCAECs transfected with different cofilin2 constructs. Experiments were repeated at least 3 times and the data are shown as mean ± SEM (*n* = 6 mice per group). *P < 0.05.

Since cofilin proteins interact with F-actin to promote actin depolymerization [40], we hypothesized whether DNA-PKcs affected actin depolymerization through cofilin. Western blots analysis in vitro showed that neither of LPS treatment nor DNA-PKcs deletion had the influences on cofilin transcription (Fig. S8A and B) and expression (Fig. 6A and B). In turn, co-immunoprecipitation (Co-IP) assays confirmed that LPS promoted the binding of cofilin1/2 to F-actin, and this crosslinking was nullified by *DNA-PKcs* ablation (Fig. 6C and D). These findings thus suggested that DNA-PKcs is required for LPS-mediated cofilin1/2-F-actin binding.

The above interactions have 2 possible explanations: one is that DNA-PKcs binds to cofilin1/2 and then promotes its interaction with F-actin; the other is that DNA-PKcs binds to F-actin as a prerequisite for the interaction between F-actin and cofilin1/2. To address these possibilities, we transfected (HA)-DNA-PKcs in human coronary artery ECs (HCAECs) prior to LPS treatment. Interestingly, HA-DNA-PKcs was preferentially pulled down by cofilin1/2, rather than by F-actin (Fig. 6E). Since there are 3 isoforms of cofilin, namely cofilin1, cofilin2, and ADF (destrin), we further explored which isoform is predominantly crosslinked by DNA-PKcs. Co-IP results showed that upon LPS treatment DNA-PKcs preferentially binds to cofilin2 (Fig. 6F to H). Indeed, no interactions between DNA-PKcs and cofilin1 or ADP were detected either in control conditions or after exposure to LPS (Fig. 6F to H). Moreover, exogenous HA-DNA-PKcs mostly interacted with cofilin2, but not with cofilin1 or ADF (Fig. S8C to E). Molecular docking simulations predicted a minimum binding energy of −11.7 kcal·mol^−1^ for the interaction between DNA-PKcs and cofilin2 (Fig. 6I to K). The potential active region for the DNA-PKcs/cofilin2 interaction and the putative hydrogen or hydrophobic bonds intervening therein are depicted in Fig. 6J and K.

We next probed the structural basis for DNA-PKcs’s interaction with cofilin2. Intriguingly, DNA-PKcs exhibits a predilection for serines or threonines succeeded by a glutamine residue (SQ/TQ motif). Consequently, we scanned this motif within the amino acid patterns of cofilin 1, cofilin 2, and ADF. No SQ/TQ motifs are present in cofilin1 or ADF (Fig. S8F and G), while an evolutionarily conserved STQ motif exists in cofilin2 (Fig. 6L). To verify whether the SQ/TQ motif of cofilin2 is implicated in its interaction with DNA-PKcs, HCAECs were alternatively reconstituted with 4 mutants of cofilin2, including one without Ser^24^ (His-cofilin2ΔS_24_), one without Thr^25^ (His-cofilin2ΔT_25_), one without Gln^26^ (His-cofilin2ΔQ_26_), and another without Thr^25^ and Gln^26^ (His-cofilin2ΔT_25_Q_26_). Co-IP assays demonstrated that loss of either T_25_ or Q_26_ motif, but not of S_24_, was able to disrupt the binding between DNA-PKcs and cofilin2 in LPS-treated cells (Fig. 6M). Similarly, upon LPS stress, improved F-actin stability (Fig. 6N and O), enhanced endothelial barrier function (Fig. 6P and Q), down-regulated adhesion molecule transcription (Fig. 6R), and increased eNOS activity (Fig. 6S) were observed in HCAECs transfected with His-cofilin2ΔT_25_Q_26_, His-cofilin2ΔT_25_, or His-cofilin2ΔQ_26_. These data indicated that LPS stimulates the binding of DNA-PKcs to cofilin2 through cofilin2’s TQ motif.

### DNA-PKcs induces actin depolymerization by phosphorylating cofilin2

**Fig. 7.**
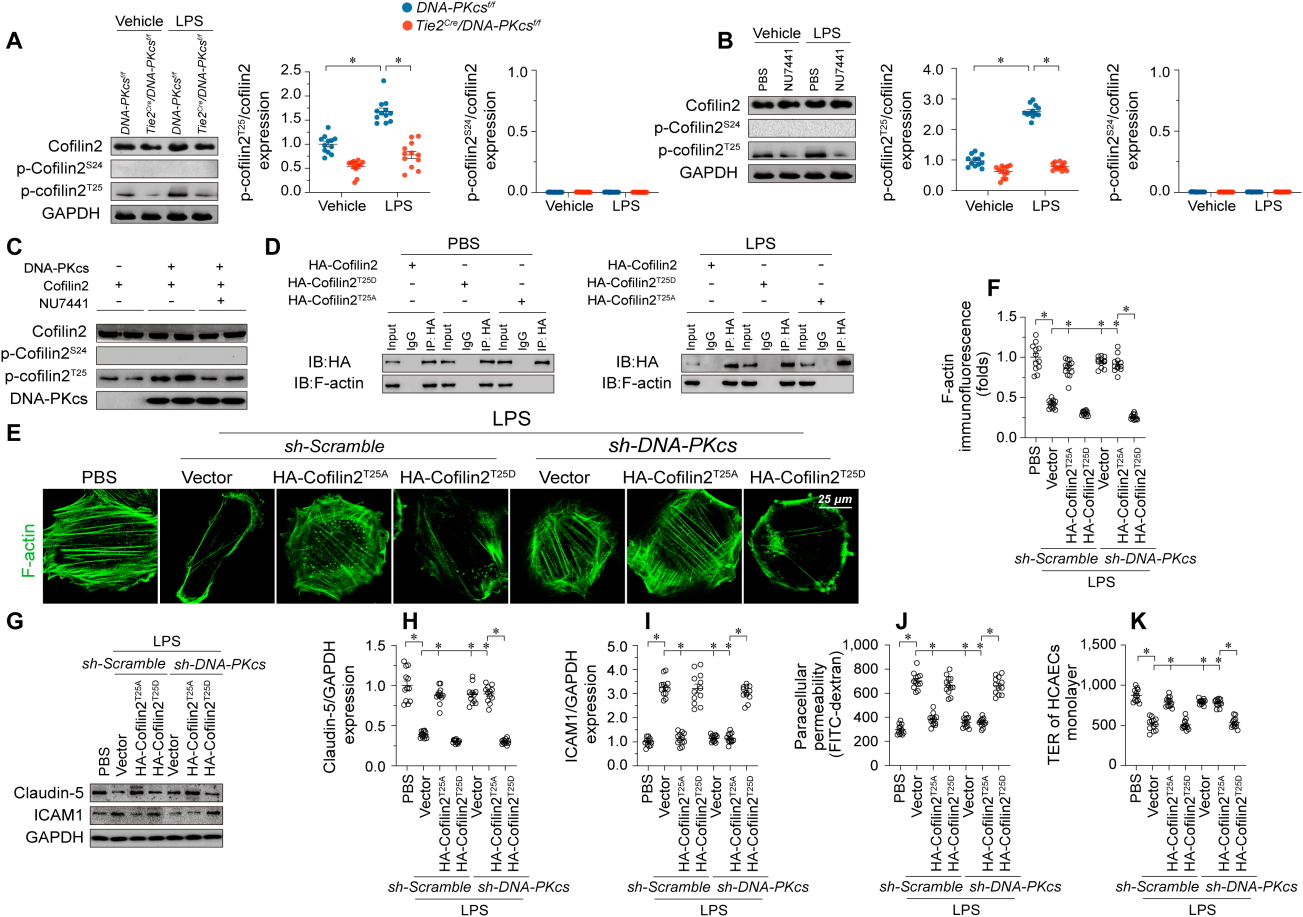
DNA-PKcs phosphorylates cofilin2 at Thr^25^. (A) Cofilin2 phosphorylation was assessed in CMECs isolated from *DNA-PKcs^f/f^*/*Tie2^Cre^* and control *DNA-PKcs^f/f^* mice in the presence of LPS. (B) Cofilin2 phosphorylation was assessed in control CMECs and NU7441-treated CMECs after LPS exposure. (C) In vitro kinase assay results in HCAECs using recombinant mouse DNA-PKcs and recombinant mouse cofilin2 proteins in the presence or absence of NU7441. The levels of cofilin2^Thr25^ phosphorylation and DNA-PKcs were determined by western blotting. (D) HCAECs were transfected with phospho-mimetic (His-cofilin2^T25D^) or phospho-defective (His-cofilin2^T25A^) cofilin2 variants. HA-tagged immunoprecipitates were collected and immunoblotted to determine the interaction between HA-cofilin2 and F-actin. (E and F) Immunofluorescence imaging of F-actin in HCAECs transfected with His-cofilin2^T25D^ and His-cofilin2^T25A^ and treated with LPS. Scale bar, 45 μm. (G to I) Western blot analysis of claudin-5 and ICAM1 expression in HCAECs transfected with His-cofilin2^T25D^ and His-cofilin2^T25A^ and treated with LPS. (J and K) Endothelial barrier function was determined by FITC-dextran clearance assays and TER detection in HCAECs transfected with His-cofilin2^T25D^ and His-cofilin2^T25A^ and treated with LPS. Experiments were repeated at least 3 times. Data are shown as mean ± SEM (*n* = 6 mice or 3 independent cell isolations per group). *P < 0.05.

Previous studies [41] have found that cofilin1/2 phosphorylation is a prerequisite for cofilin/F-actin interaction and actin depolymerization. Therefore, we asked whether DNA-PKcs-dependent cofilin2 phosphorylation triggers F-actin depolymerization. Since Ser^24^ and Thr^25^ are 2 potential DNA-PKcs phosphorylation targets in cofilin2, we designed specific antibodies targeting p-cofilin2^Ser24^ and p-cofilin2^Thr25^. After exposure to LPS, assays in CMECs isolated from control *DNA-PKcs^f/f^* mice showed that cofilin2 was phosphorylated at Thr^25^ rather than Ser^24^ (Fig. 7A). *DNA-PKcs* deletion (Fig. 7A) or inactivation (NU7441 treatment) (Fig. 7B) prevented LPS-mediated cofilin2 phosphorylation at Thr^25^ rather than Ser^24^. Consistent with these findings, in vitro kinase assays further verified that DNA-PKcs induced cofilin2 phosphorylation at Thr^25^, but not at Ser^24^, and that this effect was nullified by NU7441 (Fig. 7C).

To assess the impact of DNA-PKcs-mediated cofilin2 phosphorylation on F-actin depolymerization, we generated 2 cofilin2 mutant constructs: His-cofilin2^T25D^ (with phosphomimetic activity) and His-cofilin2^T25A^ (a nonphosphorylatable cofilin2 form). After transfection, and in the presence or absence of LPS stress, F-actin was preferentially pulled-down by His-cofilin2^T25D^ compared with His-cofilin2^T25A^ (Fig. 7D), suggesting that phosphorylated cofilin2 has increased affinity for F-actin. Confirming that LPS-mediated F-actin disassembly depends on cofilin2^Thr25^ phosphorylation, transfection of cofilin2^T25A^, but not of His-cofilin2^T25D^, prevented LPS-mediated F-actin degradation (Fig. 7E and F). Moreover, during LPS exposure, *DNA-PKcs* knockout was unable to stabilize F-actin in cells transfected with His-cofilin2^T25D^ (Fig. 7E and F).

Next, we investigated whether cofilin2 phosphorylation accounted for DNA-PKcs-mediated endothelial dysfunction upon LPS stress. Western blotting showed that LPS-mediated Claudin-5 down-regulation could be reversed by His-cofilin2^T25A^ (Fig. 7G and H), an effect accompanied by increased ICAM1 expression (Fig. 7G and I), decreased FITC-dextran permeation (Fig. 7J) and increased TER (Fig. 7K). Conversely, in HCAECs transfected with His-cofilin2^T25D^, sh-DNA-PKcs failed to prevent LPS-induced Claudin-5 down-regulation (Fig. 7G and H), ICAM1 up-regulation (Fig. 7G and I), FITC-dextran permeation (Fig. 7J), and TER decline (Fig. 7K). Similarly, His-cofilin2^T25A^ attenuated LPS-induced proinflammatory factor transcription (Fig. S9A to C). In contrast, transfection with His-cofilin2^T25D^ abrogated the anti-inflammatory effect of *DNA-PKcs* deletion in HCAECs (Fig. S9A to C). Moreover, eNOS activity was maintained by His-cofilin2^T25A^ in LPS-treated HCAECs, while transfection of His-cofilin2^T25D^ was associated with a drop in eNOS activity in sh-DNA-PKcs-expressing cells (Fig. S9D). These findings corroborated that DNA-PKcs-dependent phosphorylation of cofilin2 at Thr^25^ mediates F-actin disassembly and consequent dysfunction in cardiac ECs.

### Repression of cofilin2^Thr25^ phosphorylation preserves barrier function, decreases the inflammatory response, and restores vasodilation in LPS-challenged myocardial microvessels

**Fig. 8.**
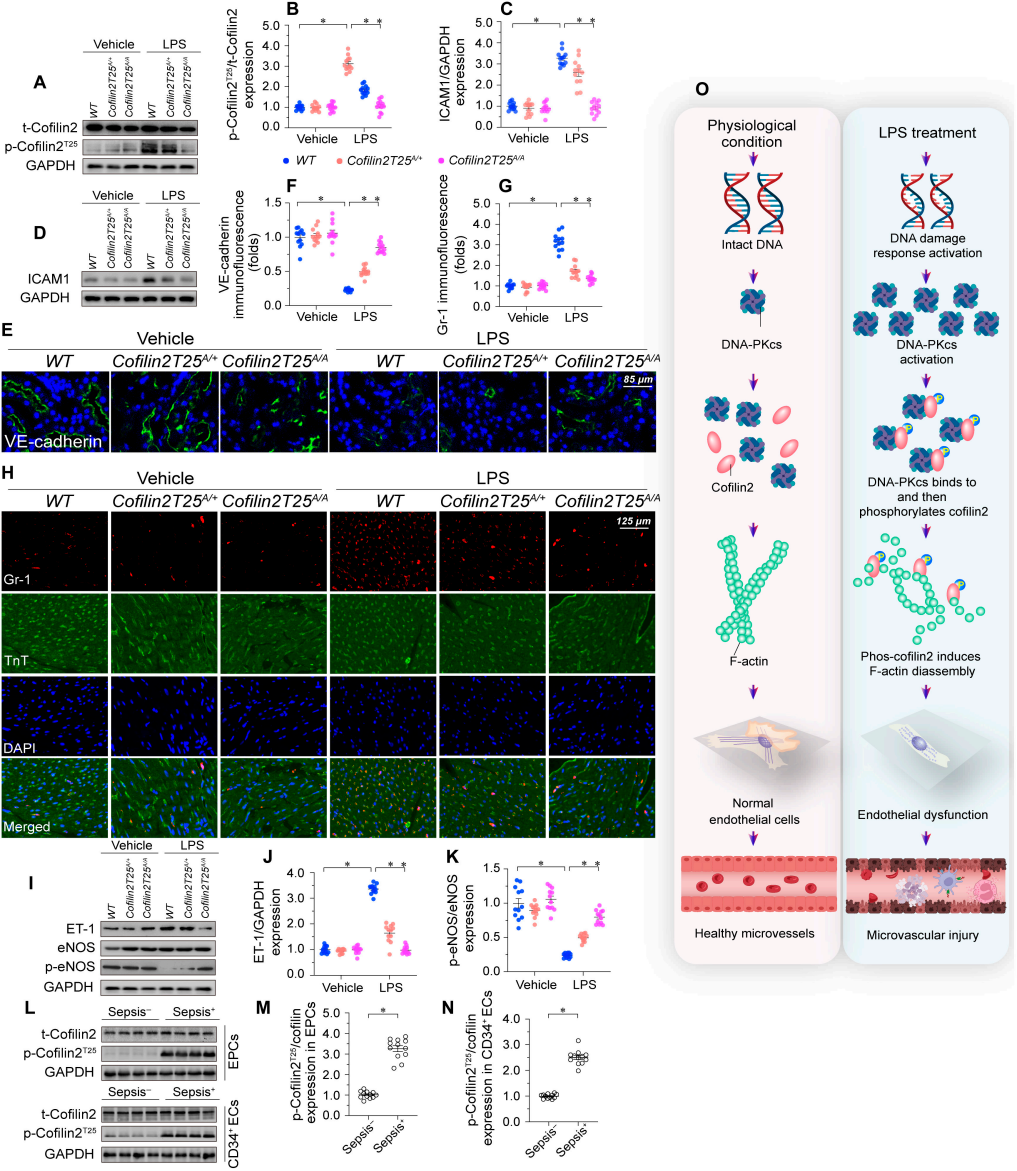
Repression of cofilin2^Thr25^ phosphorylation confers protection against endotoxemia-induced myocardial microvascular injury. WT, heterozygous *cofilin2T25^A/+^*, and homozygous *cofilin2T25^A/A^* mice were injected with LPS to model endotoxemic cardiomyopathy (*n* = 6 mice/group). (A and B) Western blot analysis of cofilin2 phosphorylation in CMECs isolated from mice. (C and D) Western blot analysis of ICAM1 in WT, heterozygous *cofilin2T25^A/+^*, and homozygous *cofilin2T25^A/A^* mice. (E and F) Immunofluorescence of VE-cadherin in myocardial microvessels. (G and H) Immunofluorescence of Gr-1^+^ neutrophils in WT, *cofilin2T25^A/+^*, and *cofilin2T25^A/A^* mice. DAPI was used to stain nuclei and TnT to stain cardiomyocytes. Scale bar, 65 μm. (I to K) Western blot analysis of p-eNOS and ET-1 expression in cardiac microvessels from WT, *cofilin2T25^A/+^*, and *cofilin2T25^A/A^* mice. (L to N) Western blots was used to evaluate cofilin2 phosphroyaltion in human circulating CD34^+^ ECs and EPCs. Experiments were repeated at least 3 times. Data are shown as mean ± SEM (*n* = 6 mice or 3 independent cell isolations per group). *P < 0.05. (O) LPS activates DNA-PKcs which ecognizes a TQ motif in cofilin2 and consequently induces cofilin2 phosphorylation at Thr^25^. Phosphorylated cofilin2 shows increased affinity for F-actin and promotes F-actin depolymerization, leading to disruption of the endothelial barrier integrity, microvascular inflammation, and defective eNOS-dependent vasodilation.

To substantiate the cellular observations delineated previously, we engineered cofilin2-knockin mice where the Thr^25^ was replaced by alanine (T25A). Pursuing this, we backcrossed heterozygous mice bearing the cofilin2 T25A knockin mutation to a C57BL/6 lineage, subsequently obtaining homozygous specimens from heterozygous pairings. Both heterozygous *cofilin2T25^A/+^* and homozygous *cofilin2T25^A/A^* mice manifested as viable, fertile, and normotypic in stature, with an absence of discernible anatomical, behavioral, or organ-specific irregularities (Fig. S10A to I). At baseline, the expression levels of phosphorylated cofilin2 (p-cofilin2^T25^) in CMECs from WT, *cofilin2T25^A/+^*, and *cofilin2T25^A/A^* mice were analogous (Fig. 8A and B). Furthermore, baseline evaluations revealed no perceptible deviations in barrier function (Fig. 8C to F), inflammatory markers (Fig. 8G and H), or vasodilation attributes (Fig. 8I to K) in *cofilin2T25^A/+^*, and *cofilin2T25^A/A^* mice. However, upon administration of LPS, a significant increase in cofilin2 phosphorylation at Thr^25^ was observed in CMECs derived from WT mice, a moderate attenuation was observed in CMECs from *cofilin2T25^A/+^* mice, and no detectable phosphorylation was observed in CMECs from *cofilin2T25^A/A^* mice. (Fig. 8A and B).

After LPS exposure, ICAM1 expression was obviously increased in WT mice, relatively reduced in *cofilin2T25^A/+^* mice, and mostly suppressed in *cofilin2T25^A/A^* mice (Fig. 8C and D). Concomitantly, VE-cadherin expression in myocardial microvessels was markedly suppressed in WT mice, partly sustained in *cofilin2T25^A/+^* mice, and markedly stabilized in *cofilin2T25^A/A^* mice (Fig. 8E and F). In line with these findings, LPS-mediated cardiac neutrophil deposition was noticeable in WT mice, partly prevented in *cofilin2T25^A/+^* mice, and largely inhibited in *cofilin2T25^A/A^* mice (Fig. 8G and H). Moreover, after LPS treatment, decreased p-eNOS and increased ET-1 expression were noted in myocardial microvessels from WT mice (Fig. 8I to K). Once again, these changes were partially reversed in *cofilin2T25^A/+^* mice and substantially prevented in *cofilin2T25^A/A^* mice (Fig. 8I to K). Lastly, the survival rate of mice was partly increased in *cofilin2T25^A/+^* mice and markedly improved in *cofilin2T25^A/A^* mice (Fig. S10J). These data suggested that phosphorylation of cofilin2 at Thr^25^ is a key determinant of LPS-mediated microvascular dysfunction in the heart.

To evaluate these findings from a clinical viewpoint, we analyzed cofilin2 phosphorylation in circulating CD34^+^ cells and EPCs from peripheral blood of septic patients and healthy controls via western blots. In both cell populations, p-cofilin2^T25^ expression was up-regulated by sepsis (Fig. 8L to N). Similar to DNA-PKcs up-regulation, and suggesting the clinical relevance of cofilin2 in endotoxemia-related organ dysfunction, in septic patients cofilin2 phosphorylation was associated with increased APACHE II an SOFA scores, decreased LVEF, reduced cardiac index, and hyperlactatemia (Table S2).

## Discussion

The results of the present study indicated that DNA-PKcs exacerbates endotoxemia-related endothelial damage and cardiac microvascular dysfunction by inducing cofilin2 phosphorylation. Three main findings can be derived from our data (Fig. 8O): First, DNA-PKcs activation is deleterious to the cardiac endothelium. Enhanced DNA-PKcs activation disrupted endothelial barrier function, promoted a transition toward a proinflammatory phenotype, and impaired eNOS-dependent vasodilation, resulting in microvascular and myocardial dysfunction in mice with LPS-induced endotoxemic cardiomyopathy. Second, DNA-PKcs recognizes a T^25^Q^26^ motif in cofilin2 and promotes cofilin2 phosphorylation at Thr^25^ in myocardial microvascular ECs. Third, phosphorylated cofilin2 has increased affinity for F-actin and triggers actin depolymerization; this contributes to cytoskeleton degradation and endothelial dysfunction, adversely affecting microvascular integrity. Thus, our results identified DNA-PKcs as a specific initial signal for endothelial dysfunction during endotoxemic cardiomyopathy. With supportive evidence provided by preclinical studies assessing the efficacy of DNA-PKcs inhibition for cancer treatment [42,43], we thus propose that therapeutic strategies that target the DNA-PKcs/cofilin2 interaction, and/or promote cytoskeleton stabilization, may be valuable to prevent or treat endotoxemia-related myocardial microvascular injury.

Accumulating evidence has identified DNA-PKcs as a mediator of pathological inflammation responses. DNA-PKcs senses cytoplasmic DNA in aged CD4^+^ T cells and thus potentiates T cell activation during aging-related autoimmune inflammation [44]. In murine splenocytes, radiation-induced oxidative injury and inflammation response have been attributed to DNA-PKcs-dependent NF-κB activation [45,46]. In natural killer cells, DNA-PKcs triggers a proinflammatory response through the endosomal signaling pathway [47]. Moreover, IR3-dependent innate immunity is highly governed by DNA-PKcs [48]. Similarly, our previous studies in a mouse sepsis model described a decreased inflammatory response upon deletion of DNA-PKcs in heart [35] or kidney [34]. The mechanistic underpinnings by which DNA-PKcs discerns and mediates proinflammatory signals may hinge on its capacity to detect cytoplasmic DNA. Historically identified as a sentinel for DNA double-strand breaks, DNA-PKcs’s role in recognizing cytoplasmic DNA emerges from evidence that such leakage is a definitive consequence of LPS-induced cellular damage across diverse tissues [49–51]. This aligns with the hypothesis that DNA-PKcs acts as an intracellular stress sensor, responding to inflammatory insults inflicted by LPS or comparable stressors. Further, oxidative stress [52] and nutrient deprivation [53] are also postulated to trigger DNA-PKcs activation. Our investigations reveal that LPS administration precipitates dose-dependent DNA damage, as indicated by the up-regulation of DNA double-strand break markers, which in turn appears to catalyze DNA-PKcs activation.

A main feature of DNA-PKcs substrates is the presence of an S/T-Q cluster domain (SCD), which contains motifs with Ser/Thr followed by a glutamine [54]. Cofilin2 contains an evolutionarily conserved STQ motif, which makes it a potential substrate of DNA-PKcs. Here, we demonstrated that Thr^25^ in cofilin2 is a target of DNA-PKcs upon LPS exposure. Compared with its unphosphorylated form, p-cofilin2^Thr25^ has increased affinity to F-actin. Similar to cofilin1 phosphorylation [41], p-cofilin2^Thr25^ causes F-actin depolymerization into G-actin to promote actin cytoskeleton degradation. These findings identified cofilin2 phosphorylation and F-actin disassembly as novel downstream effectors of DNA-PKcs activation in endotoxemia-induced endothelial injury, thus elucidating the molecular basis by which LPS promotes endothelial cytoskeleton degradation. Most previous studies focused on the impact of cofilin1 phosphorylation on endothelial dysfunction during physiopathological and toxicological conditions [55–57]. Cofilin1 is mainly expressed in nonmuscle tissue, while cofilin2 is abundant in muscle [58]. In fact, cofilin1 and ADF are predominantly expressed in embryonic muscle [59,60]; however, their levels decline during muscle development, with a progressive rise in the expression of cofilin2 [61,62]. In turn, cofilin2 is expressed in various tissues but to lower levels than in muscle tissue [63]. Although cofilin1 and cofilin2 proteins have 81% sequence homology and share all known functional domains, our findings suggest that cofilin2 in particular is a key contributor to LPS-induced endothelial damage.

Recent studies have revealed an intricate connection between DNA-PKcs activation and the inflammatory response. Wu et al. initially discovered that knockdown of *DNA-PKcs* markedly reduces the expression of TLR1, TLR3, and TLR8, suggesting that DNA-PKcs could be an inducer of the TLR signaling pathway [36]. Furthermore, Heo et al. found that LPS induces mitochondrial DNA double-strand breaks (mtDSBs), which strongly activates DNA-PKcs and subsequently triggers a type I interferon response [37]. Building upon this discovery, they concluded that inhibiting DNA-PKcs activation could serve as a safeguard against persistent inflammatory reactions. Consistent with these findings, our results confirmed that LPS up-regulates the expression of TLR4, NLRP3, and caspase-1. Interestingly, deletion of *DNA-PKcs* prevented the up-regulation of inflammatory markers in ECs, leading to a moderate reduction in the immune reaction in heart tissues. However, it is worth noting that ECs are not the primary functional cells within the myocardium. Therefore, the impact of *DNA-PKcs* deletion in ECs on the immune response in the myocardium may be limited. Consequently, the observed beneficial effects of endothelial-cell-specific *DNA-PKcs* deletion on immune reactions in the myocardium may be attributed to 2 potential mechanisms: improved endothelial barrier function and a balanced immune system in the myocardium. Importantly, a more appropriate evaluation of the contribution of DNA-PKcs-dependent innate immune reactions to pathological changes in the myocardium may be achieved using mice with *DNA-PKcs* deficiency specifically in cardiomyocytes.

Numerous paradigms have been postulated to elucidate the interplay between actin cytoskeleton depolymerization and endothelial perturbation. Critical to endothelial barrier integrity, adherens junctions materialize with the support of lamellipodia, characterized by their F-actin scaffolding [64,65]. ICAM1, an intercellular adhesion molecule, orchestrates the transendothelial navigation of leukocytes [66]. Within regions rich in F-actin, ICAM1 undergoes internalization and, contingent on F-actin, translocates to the basal plasma membrane via caveolae [67]. It was also reported that phosphorylation of cofilin1 increases nuclear translocation and activation of NF-κB, leading to ICAM1 transcription in ECs [26]. eNOS plays an indispensable role in vasodilation. Intriguingly, G-actin, derived from F-actin disassembly, emerges as a predominant constituent of a 51-kDa ribonucleoprotein. This complex associates with the eNOS mRNA 3’ untranslated domain, thereby inhibiting eNOS translation in ECs [68]. A heightened F/G-actin ratio was concomitant with augmented eNOS transcription and expression [68]. Furthermore, F-actin disassembly precedes a surge in ROS production, which subsequently attenuates eNOS activity [69]. This investigation accentuates the pivotal role of F-actin stabilization and cytoskeletal equilibrium in modulating barrier fidelity, inflammation mediation, and eNOS-driven vasodilation during endotoxemia-induced microvascular aberrations. Thus, preservation of F-actin stability represents a valid endothelial-specific protective strategy against myocardial microvascular injury during endotoxemia.

Our research corroborates that LPS-induced activation of DNA-PKcs is a pivotal factor in endothelial dysfunction during endotoxemia. The therapeutic potential of targeting DNA-PKcs activation, although promising, necessitates further empirical substantiation through clinical trials. Precedent studies have demonstrated that genetic ablation of DNA-PKcs in murine models, or the application of NU7441, a DNA-PKcs-specific inhibitor, ameliorates metabolic dysfunctions associated with obesity [70]. Additionally, in models of chronic kidney disease, deletion of DNA-PKcs in renal tubular cells or treatment with NU7441 has shown efficacy in mitigating renal fibrosis [71]. In parallel, DNA-PKcs disruption in vascular smooth muscle cells attenuates angiotensin II-induced vasodilatory dysfunction and hypertension, primarily by inhibiting vascular remodeling [72]. These observations collectively infer that prolonged DNA-PKcs inhibition may confer salutary effects on obesity, chronic renal injury, and hypertension. Nevertheless, our study elucidates that in the context of acute endotoxemia-induced vascular impairment, the long-term benefits of DNA-PKcs inhibition on endothelial integrity and vascular homeostasis remain to be established. Emerging evidence indicates a role for DNA-PKcs in mediating tissue fibrosis [71,72]. It is crucial to underscore that initial DNA-PKcs activation correlates with the loss of functional ECs, thereby impacting endothelial integrity and viability, as demonstrated in our findings. Subsequently, DNA-PKcs activation does not facilitate the regeneration or proliferation of functional ECs; rather, it contributes to fibroblast proliferation and collagen synthesis [71,72], potentially leading to the deterioration of the vascular bed. Consequently, several studies advocate that impeding DNA-PKcs-mediated tissue fibrosis could be instrumental in preventing the progression of renal dysfunction and hypertension [71,72]. In summary, DNA-PKcs activation may precipitate the loss of functional cells during acute injury and foster the proliferation of nonfunctional cells in the chronic phase of tissue repair.

In conclusion, we report a previously unknown role of DNA-PKcs as mediator of cofilin2 Thr^25^ phosphorylation and subsequent actin degradation and endothelial dysfunction in CMECs during endotoxemia. These findings may guide the development of endothelium-specific therapeutic treatments against microvascular injury in patients with endotoxemic cardiomyopathy.

## Materials and Methods

### Animals

Experimental procedures and animal care adhered strictly to protocols sanctioned by the Institutional Animal Care and Use Committee at the Chinese PLA General Hospital. We produced homozygous *DNA-PKcs^f/f^* mice as detailed previously [64]. *Tie2^Cre^* transgenic specimens (B6.Cg-Tg(Tek-cre)1Ywa/J, stock no: 008863) were procured from the Jackson Laboratory (Bar Harbor, ME, USA). By interbreeding *DNA-PKcs^f/f^* with *Tie2^Cre^* mice, we established endothelial-cell-specific *DNA-PKcs* knockout variants (*DNA-PKcs^f/f^*/*Tie2^Cre^*), which were subsequently backcrossed to a C57BL/6J lineage for 10 generations. *Cofilin2T25^A^*-knockin specimens, based on a C57BL/6 lineage, were developed by Cyagen Biosciences (Suzhou, China). Mice were nurtured in aseptic conditions, observed a 12-h light/dark regime, and enjoyed unrestricted water and standard feed access. WT C57BL/6J specimens were sourced from the Chinese PLA General Hospital Animal Center (Beijing, China).

### Endotoxemia model

Based on our recent reports[73] endotoxemia was elicited in 8-week-old male WT mice, *DNA-PKcs^f/f^*/*Tie2^Cre^* mice, and control *DNA-PKcs^f/f^* mice using an intraperitoneal injection with 10 mg/kg LPS. The animals were sacrificed 72 h later for downstream analyses. Cardiac tissue was expeditiously procured, either snap-frozen in liquid nitrogen or preserved in 4% PFA, while and sera were collected for ELISA. To inhibit the activity of DNA-PKcs, mouse was Intraperitoneally injected with NU7441 (2 mg/kg, Selleck, Catalogue: S7409) 3 d before LPS treatment.

### Ethics and human sample procurement

Ethical clearance for our human-centric investigation was granted by the Ethics Committee at the Chinese PLA General Hospital (Beijing, China; Approval No. 2018-813). Prior to enrolment, comprehensive informed consents were obtained from all participating patients. Diagnosis of septic cardiomyopathy was made based on an acute diminution in the LVEF accompanied by ventricular dilation during septic episodes [74]. Accordingly, blood specimens were sourced from both septic cardiomyopathy-afflicted patients (*n* = 66) and those absent of the condition (*n* = 174) within intensive care facilities. A detailed breakdown of patient demographics is presented in Table S1. The isolation of CD34^+^ ECs and EPCs was achieved utilizing magnetic polystyrene beads, which were cloaked with a specific monoclonal antibody, in alignment with established protocols [75].

### Comet assay and MTT

DNA damage in cultured CMECs was assessed via the comet assay. After specified treatments, CMECs were washed with cold PBS and processed using the comet assay kit (Trevigen, cat. no. 4250-050K) under alkaline conditions. DNA damage was quantified by measuring mean tail moments for 300-500 cells/sample, utilizing the Comet Assay Software Project (v1.2.3b1). The MTT assay was conducted as previously described [76].

### Cell culture

Primary mouse CMECs were isolated from *DNA-PKcs^f/f^* and *DNA-PKcs^f/f^*/*Tie2^Cre^* mice (aged 2 wk) as previously described by us [77]. Human coronary artery ECs (HCAECs) were purchased from the National Infrastructure of Cell Line Resource (Beijing, China). Both cell types were cultured in DMEM containing 10% FBS (both from HyClone, Logan, UT, USA). Cells with 80% to 85% confluence were incubated with 10 μg/ml LPS for 24 h. To inhibit DNA-PKcs and Ku80 activities, cells were respectively treated with NU7441 (1 μM; Cat. No. S2638, Selleck Chemicals, Houston, TX, USA) and STL127705 (10 μM; Cat. No. HY122727, MedChemExpress, USA) for 2 h before LPS treatment. To prevent and induce F-actin polymerization, CYD (15 μM; sc-201442, Santa Cruz Biotechnology, Dallas, TX, USA) and jasplakinolide (20 μM; sc-202191; Santa Cruz Biotechnology) were respectively added to cell cultures 6 h before LPS treatment.

### FITC-dextran clearance and TER assays

ECs were treated with FITC-dextran (1 mg/ml, Cat. No. 46944-100MG-F; Sigma) for 2 h, followed by cold-PBS wash. Endothelial permeability was assessed by measuring the absorbance at 450 nm using a PerkinElmer Enspire LF plate reader based on the FITC-dextran signal. For TER assays, ECs were cultured at 3 × 10^4^ cells/well in 24-well Transwell chambers (0.4 μM pore size, #3470; Corning). Electrical resistance of the barrier was determined by applying an AC signal across electrodes flanking the cellular monolayer, with measurements interpreted using the CellZscope Automated Cell Monitoring System (nanoAnalytics GmbH; Münster, Germany).

### Gelatin-ink perfusion assay and immunohistochemistry

In gelatin-ink perfusion assays, mouse chests were exposed, the left ventricle accessed with a 1.6 mm diameter syringe, and an outflow slit made in the right atrium. Mice were initially perfused with PBS (pH 7.2 to 7.4) at 37 °C, followed by a 5-min perfusion with 10% formalin at 37 °C once blood was cleared. Subsequently, a mixture of 10% India ink and 2% gelatin, heated to 40 °C, was infused until limb darkening. To confine the gelatin-ink, key circulatory exits including the right atrium and the major vessels were ligated. For mixture solidification, mice were placed ventral side up at −20 °C for 10 to 20 min. Heart sections (15 μm) were observed under an Olympus CHBS microscope.

Approximately 1-cm^3^ tissue blocks were either flash-frozen in OCT (Thermo Fisher Scientific) atop dry ice or fixed in 4% paraformaldehyde, dehydrated in 30% sucrose, and subsequently embedded in OCT. These samples were conserved at −80 °C pending sectioning. Sections (5 μm) were prepared using a cryostat and mounted on poly-L-lysine slides (Sigma Aldrich). For albumin detection, post heat-mediated antigen retrieval, sections were blocked with 2% BSA, probed with primary antibodies (1:100) overnight at 4 °C, and subsequently stained using the Vectastain ABC Kit and DAB substrate (SK-4100, Vector Labs) [78].

### Docking analysis

The 3D structures of human DNA-PKcs and cofilin2 were obtained from the AlphaFold Protein Structure Database. Autodock Vina (version 1.1.2) was utilized to predict possible binding modes between DNA-PKcs and cofilin2, the most likely binding mode was generated based on the docking solution that gave the lowest binding free energy (BFE) [79].

### Two-dimensional echocardiographic assessment

Two-dimensional echocardiographic measurements of cardiac function were carried out with GE Vivid7 and GE Vivid95 ultrasound devices in unventilated mice under isoflurane anesthesia (1.5% to 2.5% in 2L O_2_/min).

### DNA-PKcs kinase assay

GST-tagged recombinant human DNA-PKcs protein (Cat. No. ab204152, Abcam, Cambridge, UK) and His-tagged recombinant human cofilin2 protein (Abcam, No. ab111635) were used to perform in vitro kinase assays using an Universal Kinase Activity Kit (Cat. No EA004, Bio-Techne Corp., Minneapolis, MN, USA) according to the manufacturer’s instructions [80].

### Immunofluorescence

Mouse heart samples were promptly embedded in OCT and snap-frozen at −20 or −80 °C, then sectioned into 10-μm slices. For immunofluorescence, slides underwent a 15-min fixation in 10% formalin at room temperature (RT), 3 washes in PBST, and permeabilization using 0.5% Triton X-100. Following a 1-h block in 10% goat serum at RT, slides were incubated with antibodies targeting F-actin (#ab112125, Abcam), VE-cadherin (#ab205336, Abcam), Gr-1 (#ab314120, Abcam), TnT (#ab8295, Abcam), α-SMA (#ab5831, Abcam), syndecan-1 (#ab128936, Abcam), and γ-H2AX (#ab26350, Abcam) overnight at 4 °C. Post-wash, they were labeled with appropriate fluorescent secondaries and counterstained with DAPI. Imaging was conducted on a Zeiss LSM 510 microscope.

### RNA extraction and RT-qPCR

RNA processing utilized RNase-free reagents and containers or those treated with 0.1% DEPC (Thermo Fisher, Cat. No. 4387937). Total RNA was isolated using TRIzol (Invitrogen, Cat. No. 15596018). cDNA synthesis involved reverse transcribing 500 ng of RNA with random primers (Thermo Fisher, Cat. No. SO142) and RevertAid H Minus Reverse Transcriptase (Thermo Fisher, Cat. No. EP0452). RT-qPCR was executed on a StepOnePlus system (Applied Biosystems) with the SYBR Green Master Kit (Thermo Fisher, Cat. No. A25918). Primer details are in Table S4. Each condition had 3 biological replicates. Relative gene abundance versus internal controls was assessed using the −2^ΔΔCT^ formula.

### Western blotting

Tissues and cells were lysed in RIPA buffer with protease (Thermo Fisher, Cat. No. 36978) and phosphatase inhibitors (Sigma-Aldrich, Cat. No. P5726) for 10 min on ice [81]. Lysates were centrifuged at 14,000 rpm, 15 min, 4 °C. Protein concentrations in the supernatant were gauged via the Bradford method (Bio-Rad, Cat. No. 5000113-115). After adding LDS sample buffer (Life Technologies, #NP0007) and boiling at 90 °C for 10 min, proteins (20 μg) underwent 10% to 15% SDS-PAGE and were transferred to 0.2-μm nitrocellulose membranes (Carl Roth, Cat. No. 4685.1). Membranes, post-blocking with 5% milk for 1 h, were probed with primary antibodies overnight at 4 °C, followed by secondary antibodies for 1 h. Bands, after TBST washing, were visualized using Pierce ECL Plus substrate (Life Technologies, Cat. No. 32132). Antibody details are in Table S5.

### ELISA

ELISA kits were purchased from MyBioSource (Mouse BNP ELISA Kit, Cat. No. MBS2510603; Mouse Troponin T ELISA Kit, Cat. No. MBS7256410; Mouse Lactate Dehydrogenase ELISA Kit, Cat. No. MBS034493; Mouse Creatine Kinase MB Isoenzyme [CKMB] ELISA Kit, Cat. No. MBS2020866), LifeSpan BioSciences, Inc. (Mouse PRKDC/DNA-PKcs [Sandwich ELISA] Kit, Cat. No. LS-F8115), and Abcam (Mouse eNOS ELISA Kit, Cat. No. ab230938). Plates received standard solutions and samples (50 μl) and were incubated at RT for 2 h with shaking, followed by a quintuple wash with 200 μl of buffer. Biotinylated albumin antibody (50 μl) was then added for 1 h, succeeded by washing and a 30-min incubation with 50 μl streptavidin-peroxidase. After further washing, 50 μl of chromogen substrate was added for 30 min. Color development prompted the addition of 50-μl stop solution. Absorbance was assessed at 450 nm [82]. Protein concentrations were interpolated from a standard curve.

### Co-immunoprecipitation

Co-IP methodologies were based on our prior work [34]. HCAECs were lysed in IP buffer supplemented with protease inhibitors, MG-132, and leupeptin, followed by 20 needle passes and centrifugation at 16,000 g for 10 min. The supernatant was then incubated with EZview Red Anti-Myc Affinity Gel (E6654, Sigma-Aldrich) for 16 h at 4 °C, washed with IP buffer, and eluted using 2x loading buffer at 95 °C. After centrifugation, supernatant was combined with protein solubilization buffer and subjected to SDS-PAGE and subsequent immunoblotting with appropriate antibodies.

### Measurement of endothelial-dependent vasodilation

Aortae from *DNA-PKcs^f/f^* and *DNA-PKcs^f/f^*/*Tie2^Cre^* mice were rapidly placed in cool Krebs–Henseleit (K–H) buffer. Following initial tensioning and phenylephrine (PE) contraction, aortic segments were rinsed and endothelial integrity verified with acetylcholine (Ach). Subsequent to treatment with K-H buffer laced with either LPS (10 μg/ml) or PBS for 6 h, endothelium-mediated vasodilatory reactions to Ach (10^−9^ to 10^−5^ M) or SNP (10^−10^ to 10^−6^ M) were analyzed in PE-conditioned segments. A dose-response curve for vascular relaxation was recorded using a BL-420S Biological Signal Processing System (Chengdu Techman Tech. Co., Ltd.) [83].

### Transmission electron microscopy

TEM was adapted from established methods. Heart tissue was fixed with 2.5% glutaraldehyde in 0.1 M sodium cacodylate buffer (pH 7.4) for 1 h, followed by a rinse in 100 mM phosphate buffer (CaCl_2_-free). Post-fixation was performed with 1% osmium tetroxide, and subsequent staining utilized 2% uranyl acetate in maleate buffer (pH 5.2) [84]. After dehydration through an ethanol gradient, samples were embedded in Embed812 EMS resin and polymerized overnight at 60 °C. Ultrathin 60-nm sections, prepared using a Leica Ultra Cut UCT, were placed on carbon-coated grids and counterstained with uranyl acetate and lead citrate. Visualization was conducted on a JEM-1400 plus electron microscope at 80 KV (Joel Ltd.).

### Liquid chromatography-tandem mass spectrometry

CMECs were extracts from *DNA-PKcs^f/f^*/*Tie2^Cre^* and control *DNA-PKcs^f/f^* mice were isolated and then and treated with LPS or PBS for MS analysis. Tryptic peptides were analyzed using a nanoflow LC-MS/MS on an EASYnLC 1000 system linked to a Q Exactive HF Orbitrap through a nano-electrospray ion source (Thermo Fisher Scientific) [85]. MS datasets underwent Z-score classification, and subsequent heatmap visualizations were rendered via MeV software. The Z-score quantitatively measures the number of standard deviations a data point deviates from a mean. For instance, a Z-score of 0 implies equivalence to the mean, while a Z-score of 1.0 denotes one standard deviation from it. Z-scores can be either positive, suggesting a score above the mean, or negative, indicating one below the mean.

### Cofilin2 mutant plasmid transfection

To investigate whether changes in the phosphorylation status of cofilin2 affect its binding affinity to DNA-PKcs, 4 cofilin2 mutant plasmids were commissioned to Shandong Vigene Biosciences (China): cofilin2 without Ser^24^ (His-cofilin2ΔS_24_), cofilin2 without Thr^25^ (His-cofilin2ΔT_25_), cofilin2 without Gln^26^ (His-cofilin2ΔQ_26_), and cofilin2 without Thr^25^ and Gln^26^ (His-cofilin2ΔT_25_Q_26_). The expression plasmids were transfected into HCAECs with Lipofectamine 3000 reagent (Invitrogen).

### Adenovirus transfection

DNA-PKcs Adenovirus Activation Particles (sc-423935-LAC-2) and Ku80 Adenovirus Activation Particles (sc-29384) were purchased from Santa Cruz Biotechnology. Adenovirus ector pLKO-puro encoding scrambled shRNA (sc-108080, Santa Cruz Biotechnology), *DNA-PKcs* shRNA (h) adenovirus particles (sc-35200-V, Santa Cruz Biotechnology), Ku86 shRNA (KN519524. ORIGENE), FANCD2 shRNA (m) adenovirus particles (sc-35357-V, Santa Cruz Biotechnology), or UBE2T shRNA (m) adenovirus particles (sc-106661-V, Santa Cruz Biotechnology) were transduced into 293T cells using Lipofectamine (Invitrogen) following the manufacturer’s instructions. Adenovirus-containing supernatants from 24 and 48 h post-transfection were collected and pooled. Supernatants were diluted 1:10 and supplemented with 8 mg/ml polybrene (Millipore, Burlington, MA). Cell infection proceeded for 24 h, upon which viral media was removed and replaced with DMEM.

### Statistical analysis

Data are presented as mean ± SEM (standard error of mean). All statistical analyses were performed with GraphPad Prism 8.0 (GraphPad Software Inc, USA). All sample sets were normally distributed as tested with the Shapiro-Wilk normality test. Two-tailed unpaired Student *t* test was used to compare 2 groups; 1-way analysis of variance (ANOVA) followed by Dunnett’s multiple comparisons test was used to compare differences when multiple groups were compared with a single control; one-way ANOVA followed by Tukey’s multiple comparisons test was used to compare differences when multiple groups were compared with each other; 2-way ANOVA followed by Tukey’s multiple comparisons test was used for comparisons when there were 2 independent variables. For survival curves, the Mantel–Cox test was used. Significance was defined as *P* < 0.05.

## Data Availability

The datasets used and/or analyzed during the current study are available from the corresponding author upon reasonable request.
