# Supplementary material for: DNA-PKcs Phosphorylates Cofilin2 to Induce Endothelial Dysfunction and Microcirculatory Disorder in Endotoxemic Cardiomyopathy
Source: Research (Wash D C). 2024 Mar 26;7:0331. doi: 10.34133/research.0331 (PMC10976589; doi:10.34133/research.0331)
Supplement: Supplementary 1 — Figs. S1 to S10 Tables S1 to S5 [file research.0331.f1.pdf]

DNA-PKcs phosphorylates cofilin2 to induce endothelial dysfunction and microcirculatory disorder in endotoxemic cardiomyopathy

Yingzhen Du[1#], Pingjun Zhu[1#], Yukun Li[2], Jiachi Yu[3], Tian Xia[3], Xing Chang[4], Ruibing Li[3]*, Qingyong He[4*]

[1]The Second Medical Center & National Clinical Research Center for Geriatric Diseases, Chinese PLA General Hospital, Medical School of Chinese PLA, Beijing, 100853, China
[2]Department of Cardiology, Beijing Anzhen Hospital, Capital Medical University, Beijing 100029, China
[3]Department of Clinical Laboratory Medicine, The First Medical Centre, Medical School of Chinese People's Liberation Army, Beijing, China
[4]Guang'anmen Hospital, China Academy of Chinese Medical Sciences, Beijing, 100053, China
#The first two authors contributed equally to this article.

*Corresponding Author
Ruibing Li, email: liruibing@plagh.org
Department of Clinical Laboratory Medicine, The First Medical Centre, Medical School of Chinese People's Liberation Army, Beijing, China

Qingyong He, email: heqingyongg@163.com
Guang'anmen Hospital, China Academy of Chinese Medical Sciences, Beijing, 100053, China

Supplemental Table

Supplemental Table 1. DNA-PKcs activity was analyzed by ELISA kit in EPC isolated from septic ICU patients. The association between DNA-PKcs activity and heart dysfunction was measured.

Parameters	**Low DNA-PKcs activity (n=79)**	**High DNA-PKcs activity (n=181)**	**p value**
Heart rate (bmp)	89±12	104±17	<0.001
Average arterial pressure (mmHg)	84±12	84±13	0.463
Central venous pressure (mmHg)	8.4±1.2	9.3±1.4	0.631
Ejection fraction (%)	66.5±4.3	45.2±7.6	<0.001
Mitral annular plane systolic excursion (mm)	14.5±2.9	10.9±3.3	<0.001

Tricuspid Annular Plane Systolic Excursion (mm)	20.4±3.1	15.2±3.6	<0.001
Fractional Area Change (%)	47.7±10.8	39.2±11.9	<0.001
Troponin I (μg/L)	0.03±0.01	0.27±0.09	<0.001
NT-ProBNP (ng/L)	998.5±376.8	4413.6±1755.2	<0.001
Arterial blood lactate (mmol/L)	1.7±0.6	2.5±0.8	0.004
APACHE II score	20±3	24±3	0.015
SOFA score	10.2±2.5	12.4±2.6	0.026

NT-ProBNP, N-terminal pro b-type natriuretic peptide, APACHE II, Acute Physiology and Chronic Health Evaluation II; SOFA, Sequential organ failure assessment

Supplemental Table 2. Cofilin2 phosphorylation was analyzed by Western blots in EPC isolated from septic ICU patients. The association between Cofilin2 phosphorylation and heart dysfunction was measured.

Parameters	**Low Cofilin2 phosphorylation (n=93)**	**High Cofilin2 phosphorylation (n=167)**	**p value**
Heart rate (bmp)	88±11	102±14	<0.001
Average arterial pressure (mmHg)	84±11	85±12	0.463
Central venous pressure (mmHg)	8.6±1.3	9.2±1.6	0.811
Ejection fraction (%)	62.7±5.5	47.4±6.2	0.002
Mitral annular plane systolic excursion (mm)	13.6±2.3	11.1±2.5	0.004
Tricuspid Annular Plane Systolic Excursion (mm)	20.7±3.3	16.1±2.9	<0.001
Fractional Area Change (%)	45.7±8.9	40.2±10.6	<0.001
Troponin I (μg/L)	0.06±0.03	0.23±0.10	<0.001
NT-ProBNP (ng/L)	1248.5±415.7	4197.2±1876.4	<0.001
Arterial blood lactate (mmol/L)	1.8±0.7	2.4±0.6	0.012
APACHE II score	21±3	24±3	0.028

SOFA score	10.8±2.6	12.3±2.4	0.036

NT-ProBNP, N-terminal pro b-type natriuretic peptide, APACHE II, Acute Physiology and Chronic Health Evaluation II; SOFA, Sequential organ failure assessment

Supplemental Table 3. Patient demographics for septic patients diagnosed with septic cardiomyopathy (SC) (+) or without septic cardiomyopathy (SC) (-).

Patient characteristics	SC (-) patients (n=184)	SC (+) patients (n=76)
Age (range)	64.9±15.8	77.7±15.2
Sex	Male (n=89)	Male (n=30)
Body Weight (Kg)	61.9±11.4	62.4±9.4
Comorbidities		
Atrial fibrillation (n)	8	25
Heart failure (n)	13	28
Diabetes (n)	9	34
Hypertension (n)	4	12
Tumor (n)	3	7
Coronary artery disease (n)	35	7
Clinical data		
Maximum body temperature (°C)	37.7±1.8	38.3±1.2
White blood cells ($x10^9$/L)	10.5±1.9	12.8±2.7
CRP (mg/dl)	6.5±0.9	22.7±5.5
PCT (ng/dl)	7.2±1.9	12.6±2.7
platelet ($x10^9$/L)	152.1±99.4	87.5±69.4
Lactic acid (mmol/L)	1.9±0.24	4.86±1.92
APACHE II score	19±4	27±8.2
SOFA score	7.1±2.3	13.4±3.2

CRP, C-reactive protein; PCT, procalcitonin; APACHE II, Acute Physiology and Chronic Health Evaluation II; SOFA, Sequential organ failure assessment

Supplemental Table 4. Primers for qPCR

Gene	Forward Prime	Reverse Prime
Mouse *Il-6*	5'-CAGACTCGCGCCTCTAAGGAGT-3'	5'-GATAGCCGATCCGTCGAA-3'

Mouse *Tnf-α*	5'-AGATGGAGCAACCTAAGGTC-3	5'-GATAGCCGATCCGTCGAA-3'
Mouse *Mcp1*	5'-GATAGCCGATCCGTCGAA-3'	5′-GCTACCACAACATCTGGACATT-3′
Mouse *Mmp9*	5′-AACCAATGATGCTGGGTTCAC-3′	5'-GCGCCGACTCAGAGGTGT-3'
Human *CYTB*	5′-TATCCGCCATCCCATACATT-3′	5'-GGTGATTCCTAGGGGGTTGT-3'
Human *MT-RNR1*	5′-AGCGCAAGTACCCACGTAAA-3′	5'-AGGGCCCTGTTCAACTAAGC-3'
Gapdh	5′-TCGATATTGAGCGTCCAACCT-3′	5′-CAAAGGCACGTTTGGCATACA-3′

Supplemental Table 5. Antibody information

Name	Catalogue number	Dilution factor
DNA-PKcs	Abcam, #ab32566	1:1000
p-DNA-PKcs	Abcam, #ab18192	1:1000
Ku80	Abcam, #ab80592	1:1000
Tubulin	Abcam, #ab7291	1:1000
α-SMA	Abcam, #ab5831	1:1000
Syndecan1	Abcam, #ab128936	1:1000
F-actin	Abcam, #ab205	1:1000
Albumin	Abcam, #ab192603	1:1000
JNK	Abcam, #ab307802	1:1000
p-JNK	Abcam, #ab307802	1:1000
ERK	Abcam, #ab184699	1:1000
p-ERK	Abcam, #ab192591	1:1000
GR-1	Abcam, #ab25377	1:1000
TnT	Abcam, #ab8295	1:1000
Albumin	Abcam, #ab192603	1:1000
eNOS	Abcam, #ab300071	1:1000
p-eNOS	Abcam, #ab215717	1:1000
cofilin1/2	Abcam, #ab131519	1:1000
cofilin1	Abcam, #b124979	1:1000
cofilin2	Abcam, #ab300128	1:1000

ADF/Gelsolin	Abcam, #ab109014	1:1000
VE-Cadherin	Abcam, #ab205336	1:1000
Claudin-5	Abcam, #ab131259	1:1000
ICAM1	Abcam, #ab171123	1:1000
Caspase-1	Abcam, #ab138483	1:1000
NLRP3	Abcam, #ab263899	1:1000
TLR4	Cell Signaling Technology, #ab14358	1:1000
VCAM1	Abcam, #ab271899	1:1000
MOTS-c	MyBioSource, #MBS542112	1:1000
Fibrin	Creative-biolabs, #MOB-0417ZL	1:1000

Supplemental Figures

Supplemental Figure 1

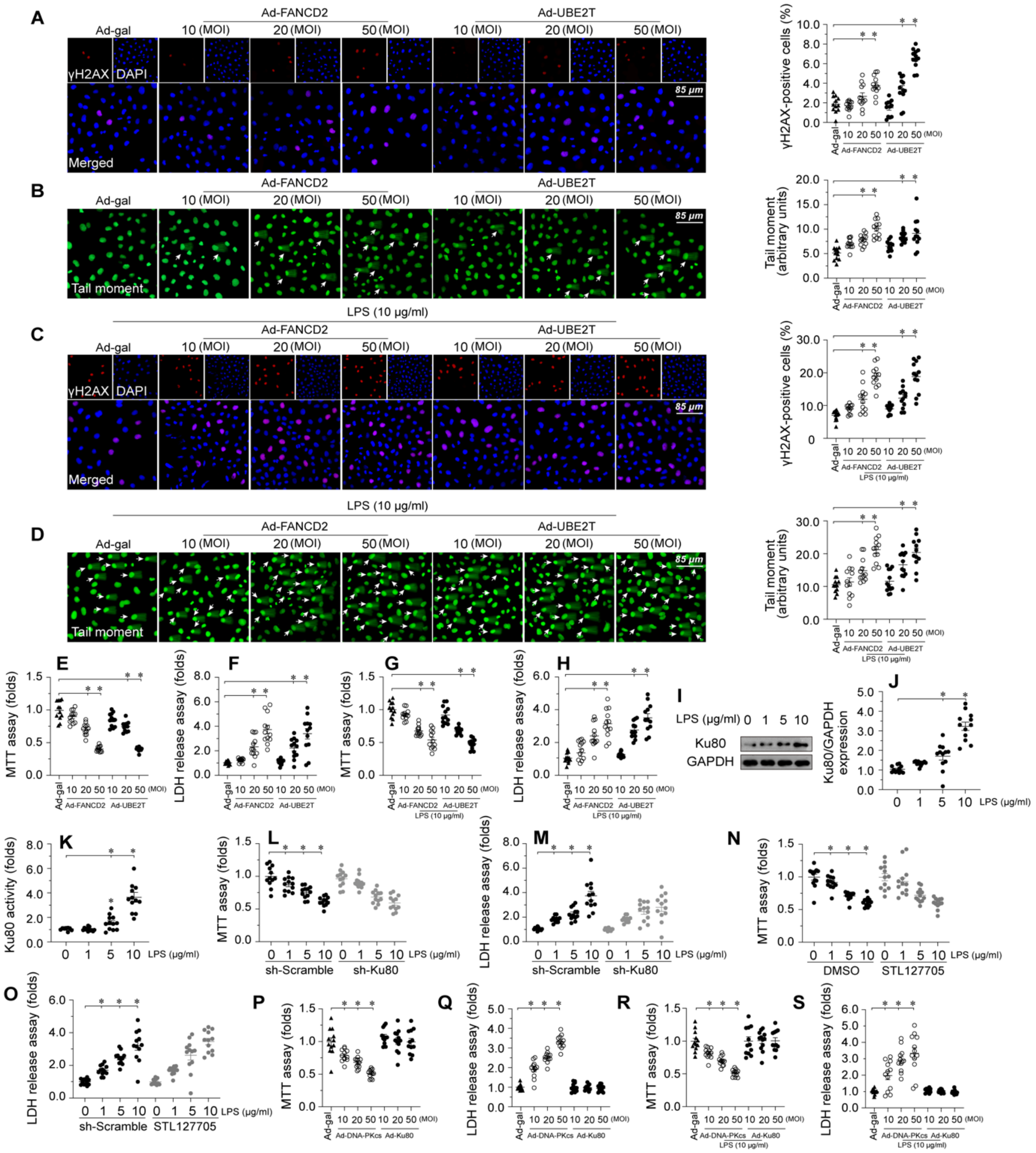

Supplementary Figure 1. Endotoxemia triggers cardiac endothelial DNA damage through DNA-PKcs. (A) Representative immunostaining and statistical data for γH2AX-positive CMECs transduced with adenovirus vectors encoding FANCD2 (Ad-FANCD2) or UBE2T (Ad-UBE2T). **(B)** DNA damage was assessed by comet assays in CMECs transduced with Ad-FANCD2 or Ad-UBE2T. The arrows indicate nuclei with DNA damage. **(C)** Representative immunostaining and statistical data for γH2AX-positive CMECs transduced with adenovirus vectors encoding FANCD2 (Ad-FANCD2) or UBE2T (Ad-UBE2T) in the presence of LPS (10 μg/ml). **(D)** DNA damage was assessed by comet assays in CMECs transduced with Ad-FANCD2 or Ad-UBE2T in the presence of LPS (10 μg/ml). The arrows

indicate nuclei with DNA damage. **(E)** MTT assay was used to evaluate cell viability in CMECs transduced with Ad-FANCD2 or Ad-UBE2T. **(F)** ELISA was used to measure LDH levels in culture media from CMECs transduced with Ad-FANCD2 or Ad-UBE2T. **(G)** MTT assay was used to evaluate cell viability in CMECs transduced with Ad-FANCD2 or Ad-UBE2T in the presence of LPS (10 μg/ml). **(H)** ELISA was used to measure LDH levels in culture media from CMECs transduced with Ad-FANCD2 or Ad-UBE2T in the presence of LPS (10 μg/ml). **(I, J)** Representative western blots and statistical data depicting changes in Ku80 expression in LPS-treated CMECs. **(K)** ELISA was used to evaluate Ku80 activity in CMECs exposed to LPS. **(L)** MTT assay was used to evaluate cell viability in CMECs transduced with sh-Ku80. **(M)** ELISA was used to measure LDH levels in culture media from CMECs transduced with sh-Ku80. **(N)** MTT assay was used to evaluate cell viability in CMECs treated with the Ku80 inhibitor STL127705. **(O)** ELISA was used to measure LDH levels in culture media from CMECs treated with STL127705. **(P)** MTT assay was used to evaluate cell viability in CMECs transduced with adenoviral vectors encoding DNA-PKcs (Ad-DNA-PKcs) or Ku80 (Ad-Ku80). **(Q)** ELISA was used to measure LDH levels in culture media from CMECs transduced with sAd-DNA-PKcs or Ad-Ku80. **(R)** MTT assay was used to evaluate cell viability in CMECs transduced with adenoviral vectors encoding DNA-PKcs (Ad-DNA-PKcs) or Ku80 (Ad-Ku80) in the presence of LPS (10 μg/ml). **(S)** ELISA was used to measure LDH levels in culture media from CMECs transduced with sAd-DNA-PKcs or Ad-Ku80 in the presence of LPS (10 μg/ml). Experiments were repeated at least three times. Data are shown as mean ± SEM (n=6 mice or three independent cell isolations per group). $*p<0.05$.

Supplemental Figure 2

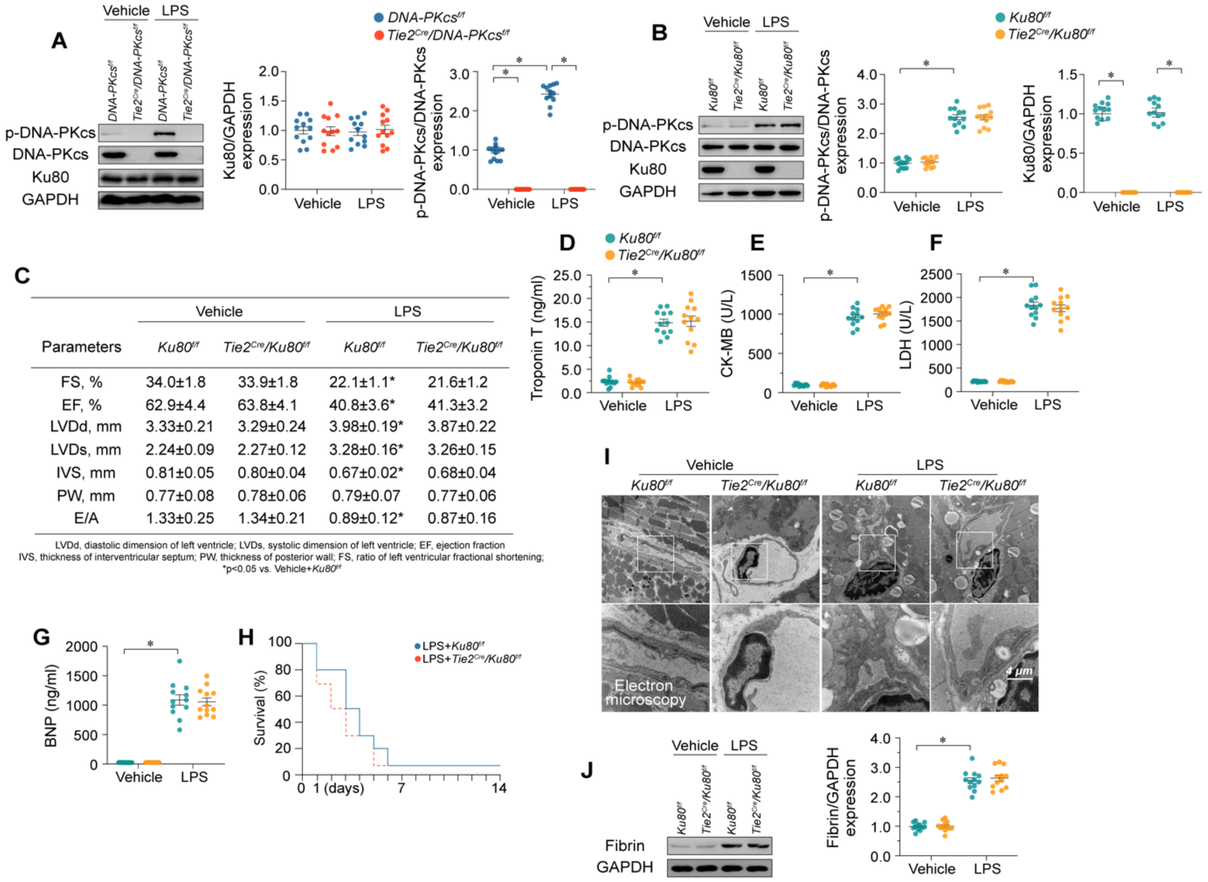

Parameters	Vehicle *Ku80^f/f*	Vehicle *Tie2^Cre/Ku80^f/f*	LPS *Ku80^f/f*	LPS *Tie2^Cre/Ku80^f/f*
FS, %	34.0±1.8	33.9±1.8	22.1±1.1*	21.6±1.2
EF, %	62.9±4.4	63.8±4.1	40.8±3.6*	41.3±3.2
LVDd, mm	3.33±0.21	3.29±0.24	3.98±0.19*	3.87±0.22
LVDs, mm	2.24±0.09	2.27±0.12	3.28±0.16*	3.26±0.15
IVS, mm	0.81±0.05	0.80±0.04	0.67±0.02*	0.68±0.04
PW, mm	0.77±0.08	0.78±0.06	0.79±0.07	0.77±0.06
E/A	1.33±0.25	1.34±0.21	0.89±0.12*	0.87±0.16

LVDd, diastolic dimension of left ventricle; LVDs, systolic dimension of left ventricle; EF, ejection fraction
IVS, thickness of interventricular septum; PW, thickness of posterior wall; FS, ratio of left ventricular fractional shortening;
*p<0.05 vs. Vehicle+*Ku80^f/f*

Supplementary Figure 2. Deletion of Ku80 fails to restore myocardial microvascular perfusion following endotoxemic stress. (A) *DNA-PKcs$^{f/f}$* mice were bred to *Tie2^{Cre}* mice to generate endothelial cell-specific *DNA-PKcs* knockout (*DNA-PKcs$^{f/f}$/Tie2^{Cre}*) mice. Septic cardiomyopathy was induced by LPS (10 mg/kg) injection. Primary mouse cardiac microvascular endothelial cells (CMECs) were isolated from mice and western blots were used to analyze the expression of p-DNA-PKcs and Ku80. **(B)** *Ku80$^{f/f}$* mice were bred to *Tie2^{Cre}* mice to generate endothelial cell-specific *Ku80* knockout (*Ku80$^{f/f}$/Tie2^{Cre}*) mice. Septic cardiomyopathy was induced by LPS (10 mg/kg) injection. Primary mouse cardiac microvascular endothelial cells (CMECs) were isolated from mice and western blots were used to analyze the expression of p-DNA-PKcs and Ku80. **(C)** Heart function was assessed by echocardiography 48 h later. **(D-G)** Serum TnT, CK-MB, BNP, and LDH levels were measured by ELISA in *Ku80$^{f/f}$/Tie2^{Cre}* and control *Ku80$^{f/f}$* mice. **(H)** Survival data for *Ku80$^{f/f}$/Tie2^{Cre}* and control *Ku80$^{f/f}$* mice. **(I)** TEM analysis of ultrastructural alterations in microvessels from *Ku80$^{f/f}$/Tie2^{Cre}* and control *Ku80$^{f/f}$* mice. **(J)** Western blot analysis of fibrin expression in cardiac microvessels from *Ku80$^{f/f}$/Tie2^{Cre}* and control *Ku80$^{f/f}$* mice. Experiments were repeated at least three times. Data are shown as mean ± SEM (n=6 mice or three independent samples per group). *p<0.05.

Supplemental Figure 3

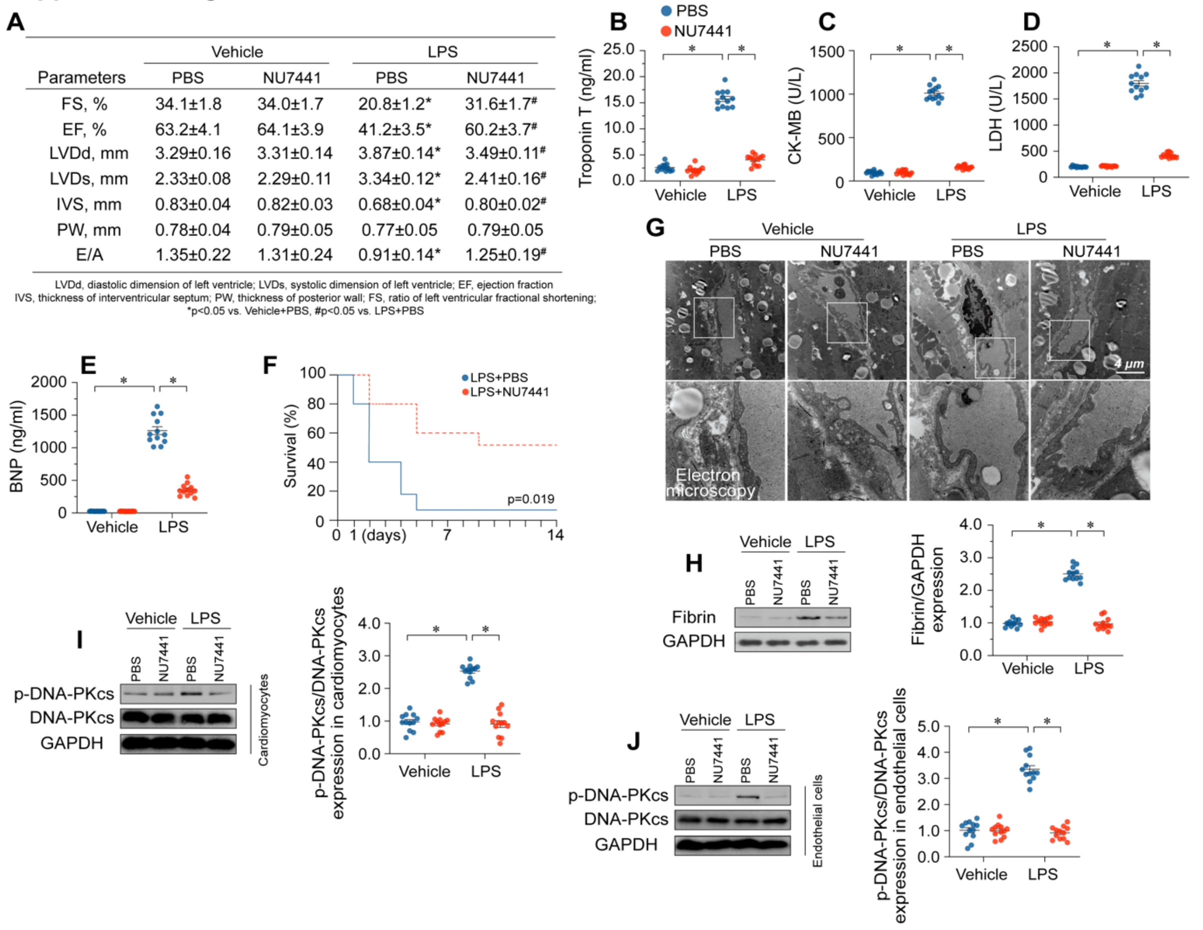

	Vehicle		LPS	
Parameters	PBS	NU7441	PBS	NU7441
FS, %	34.1±1.8	34.0±1.7	20.8±1.2*	31.6±1.7#
EF, %	63.2±4.1	64.1±3.9	41.2±3.5*	60.2±3.7#
LVDd, mm	3.29±0.16	3.31±0.14	3.87±0.14*	3.49±0.11#
LVDs, mm	2.33±0.08	2.29±0.11	3.34±0.12*	2.41±0.16#
IVS, mm	0.83±0.04	0.82±0.03	0.68±0.04*	0.80±0.02#
PW, mm	0.78±0.04	0.79±0.05	0.77±0.05	0.79±0.05
E/A	1.35±0.22	1.31±0.24	0.91±0.14*	1.25±0.19#

LVDd, diastolic dimension of left ventricle; LVDs, systolic dimension of left ventricle; EF, ejection fraction IVS, thickness of interventricular septum; PW, thickness of posterior wall; FS, ratio of left ventricular fractional shortening; *p<0.05 vs. Vehicle+PBS, #p<0.05 vs. LPS+PBS

Supplementary Figure 3. Pharmacological inactivation of DNA-PKcs preserves myocardial microvascular perfusion following endotoxemic stress. (A) NU7441 or PBS were administered to WT mice 12 h before LPS injection (10 mg/kg). Heart function was assessed by echocardiography 48 h later. **(B-E)** Serum TnT, CK-MB, BNP, and LDH levels were measured by ELISA in control and NU7441-treated mice following endotoxemia induction. **(F)** Survival data for control and NU7441-treated mice. **(G)** TEM-based detection of ultrastructural alterations in microvessels from control and NU7441-treated mice. Yellow arrows indicate endothelial swelling, microvascular wall destruction, and luminal stenosis. **(H)** Western blot analysis of fibrin expression in cardiac microvessels from control and NU7441-treated mice. **(I)** Cardiomyocytes were isolated from mice treated with NU7441 or PBS and then western blots were used to detect the expression of p-DNA-PKcs. **(J)** Endothelial cells were isolated from mice treated with NU7441 or PBS and then western blots were used to detect the expression of p-DNA-PKcs. Experiments were repeated at least three times. Data are shown as mean ± SEM (n=6 mice or three independent samples per group). *p<0.05.

Supplemental Figure 4

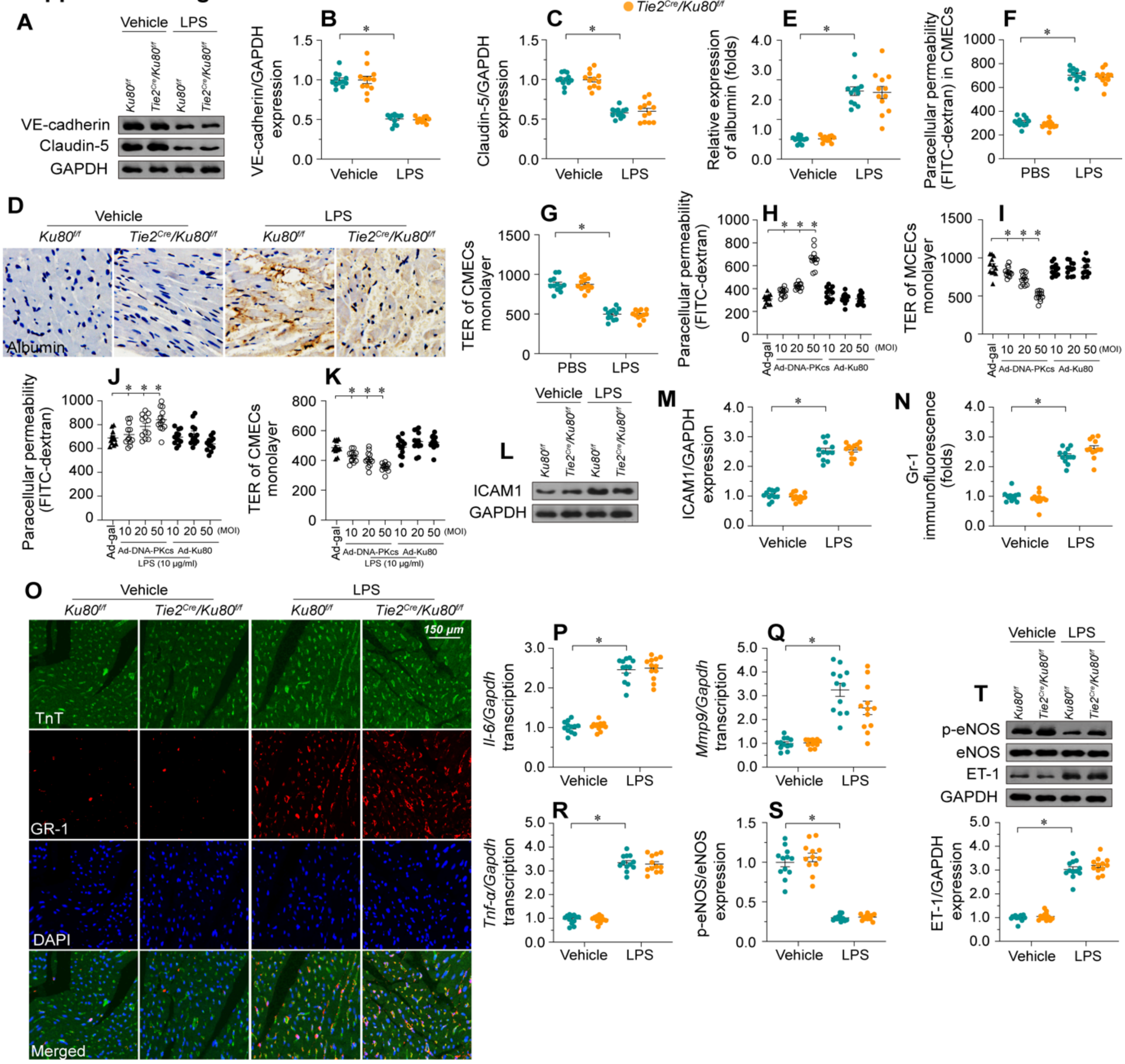

Supplementary Figure 4. Loss of Ku80 fails to prevent endotoxemia-induced barrier dysfunction, inflammation, and vasoconstriction in myocardial microvessels. (A-C) Western blot analysis of VE-cadherin and claudin-5 expression in cardiac microvessels from *Ku80$^{f/f}$*/*Tie2^{Cre}* and control *Ku80$^{f/f}$* mice treated with LPS. **(D, E)** Immunohistochemistry was used to assess albumin leakage into myocardium in LPS-treated *Ku80$^{f/f}$*/*Tie2^{Cre}* and control *Ku80$^{f/f}$* mice. **(F)** FITC-dextran clearance assays were performed in CMECs isolated from *Ku80$^{f/f}$*/*Tie2^{Cre}* and control *Ku80$^{f/f}$* mice following LPS treatment (10 μg/ml for 24 h). **(G)** TER assays were conducted in CMECs to evaluate endothelial barrier integrity. **(H)** FITC-dextran clearance assays were performed in CMECs transduced with Ad-DNA-PKcs or Ad-Ku80. **(I)** Results of TER analysis in CMECs. **(J, K)** FITC-dextran clearance assays and TER analysis were performed in CMECs transduced with Ad-DNA-PKcs or Ad-Ku80 in the presence of LPS. **(L, M)** Western blot analysis of ICAM-1 expression in cardiac microvessels from LPS-treated *Ku80$^{f/f}$*/*Tie2^{Cre}* and control *Ku80$^{f/f}$* mice. **(N, O)** Immunofluorescence of Gr-1^{+} neutrophils in heart tissues from *Ku80$^{f/f}$*/*Tie2^{Cre}* and control *Ku80$^{f/f}$* mice. DAPI was used to stain nuclei and TnT was used to stain

cardiomyocytes. **(P-R)** RT-qPCR was applied to analyze the transcription of *Mmp-9*, *IL-6*, and *Tnf-α* in cardiac tissues from *Ku80$^{f/f}$/Tie2^{Cre}* and control *Ku80$^{f/f}$* mice. **(S, T)** Western blot analysis of p-eNOS and ET-1 expression in heart tissues from *Ku80$^{f/f}$/Tie2^{Cre}* and control *Ku80$^{f/f}$* mice. Experiments were repeated at least three times. Data are shown as mean ± SEM (n=6 mice or three independent cell isolations per group). *p<0.05.

Supplemental Figure 5

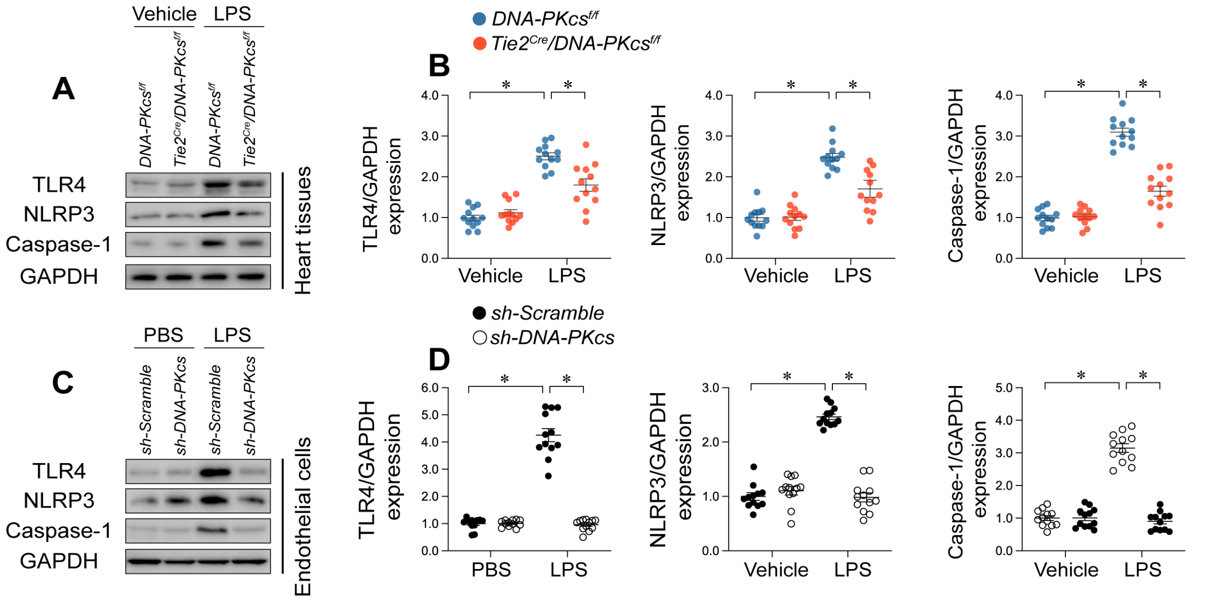

Supplementary Figure 5. Deletion of DNA-PKcs improves immune reactions. (A-B) *DNA-PKcs$^{f/f}$* mice were bred to *Tie2^{Cre}* mice to generate endothelial cell-specific *DNA-PKcs* knockout (*DNA-PKcs$^{f/f}$/Tie2^{Cre}*) mice. Septic cardiomyopathy was induced by injecting LPS (10 mg/kg), and heart tissues were isolated for western blot analysis. **(C-D).** Primary mouse cardiac microvascular endothelial cells (CMECs) were isolated from mice and transfected with shRNA against DNA-PKcs (sh-DNA-PKcs) or scramble shRNA (sh-Scramble) prior to exposure to LPS (10 μg/ml; 24 h). Western blot analysis was performed to assess the expression of TLR4, NLRP3, and caspase-3 in CMECs. Experiments were repeated at least three times, and the data are presented as mean ± SEM (three independent cell isolations). *p<0.05.

Supplemental Figure 6

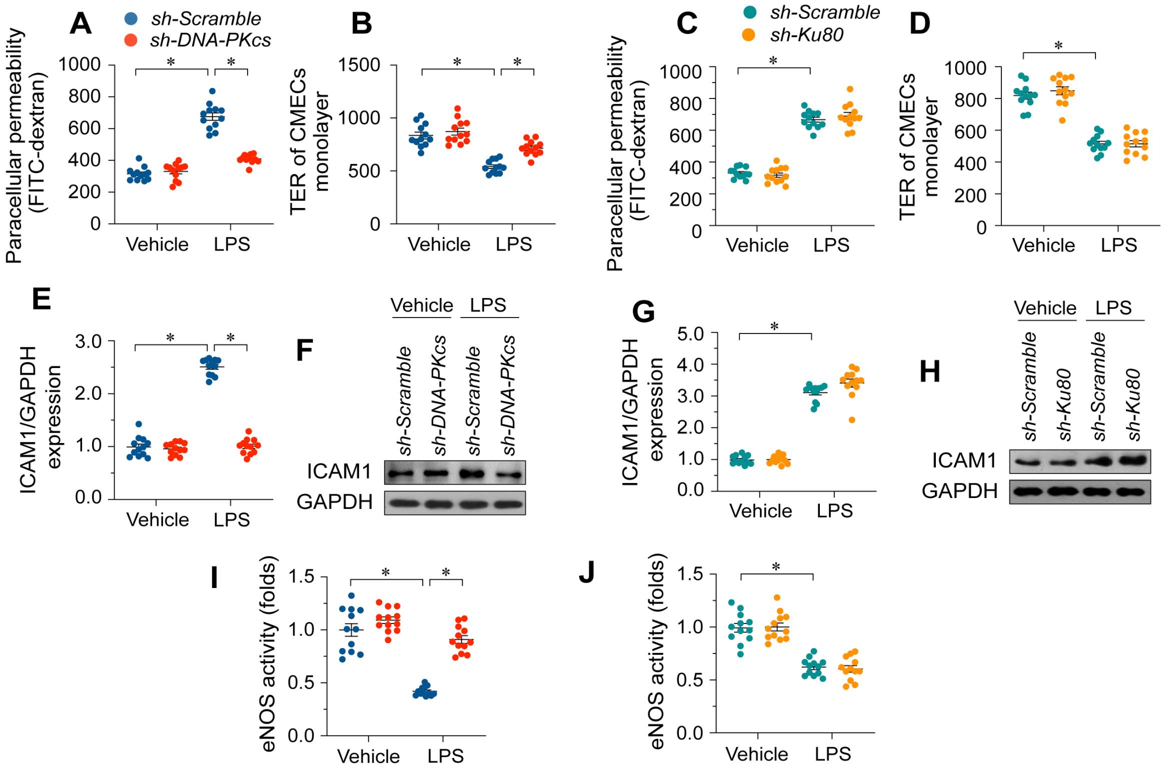

Supplementary Figure 6. Deletion of DNA-PKcs attenuates LPS-induced endothelial barrier dysfunction, adhesion molecule overexpression, and p-eNOS downregulation in circulating human EPCs. (A) FITC-dextran clearance assays were performed to evaluate the effect of LPS exposure (10 µg/ml for 24 h) on the permeability of circulating EPCs isolated from healthy subjects following transduction with shRNA against DNA-PKcs (sh-DNA-PKcs) or scramble shRNA (sh-Scramble). **(B)** TER assays were conducted in LPS-treated EPCs transfected with sh-DNA-PKcs or sh-Scramble. **(C)** FITC-dextran clearance assays were performed after LPS exposure (10 µg/ml; 24 h) in circulating human endothelial progenitor cells (EPCs) transduced with sh-Ku80 or sh-Scramble. **(D)** TER was measured in LPS-treated EPCs transduced with sh-Ku80 or sh-Scramble. **(E, F)** Western blot analysis of ICAM-1 expression in LPS-treated EPCs expressing sh-DNA-PKcs or sh-Scramble. **(G, H)** Western blot analysis of ICAM-1 expression in LPS-treated EPCs expressing sh-Ku80 or sh-Scramble. **(I)** ELISA was used to analyze the activity of eNOS in EPCs *in vitro* transfection with sh-DNA-PKcs or sh-Scramble. **(J)** ELISA was used to analyze the activity of eNOS in EPCs *in vitro* transfection with sh-Ku80 or sh-Scramble. Experiments were repeated at least three times. Data are shown as mean ± SEM (three independent cell isolations). *p<0.05.

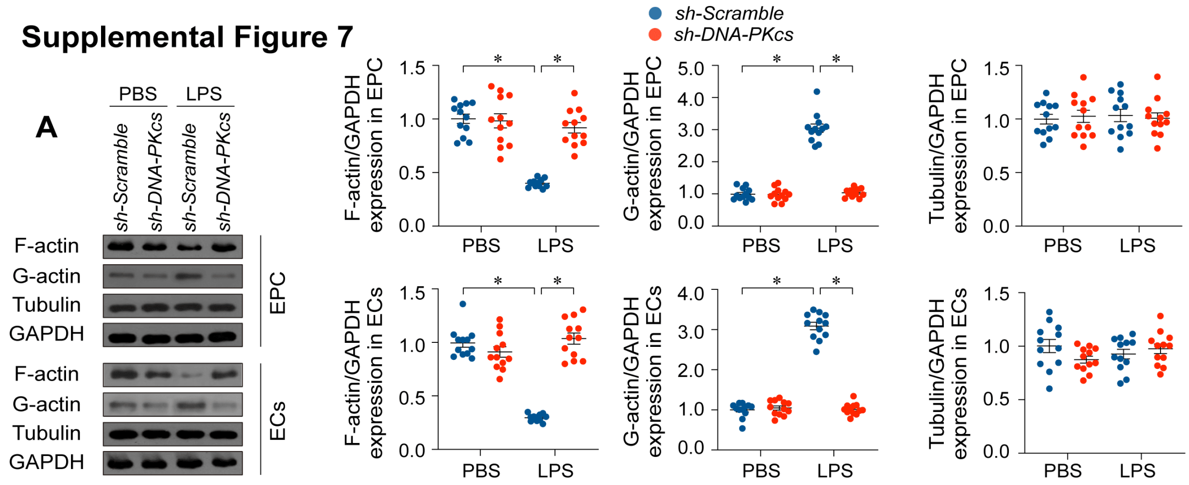

Supplementary Figure 7. DNA-PKcs depletion regulates the expression of cytoskeleton-related proteins in circulating human CD34$^+$ ECs and EPCs. (A) Western blot analysis of F-actin and G-actin expression in CD34$^+$ ECs or EPCs *in vitro* transfection with sh-DNA-PKcs or sh-Scramble. Experiments were repeated at least three times. Data are shown as mean ± SEM (three independent cell isolations). *p<0.05.

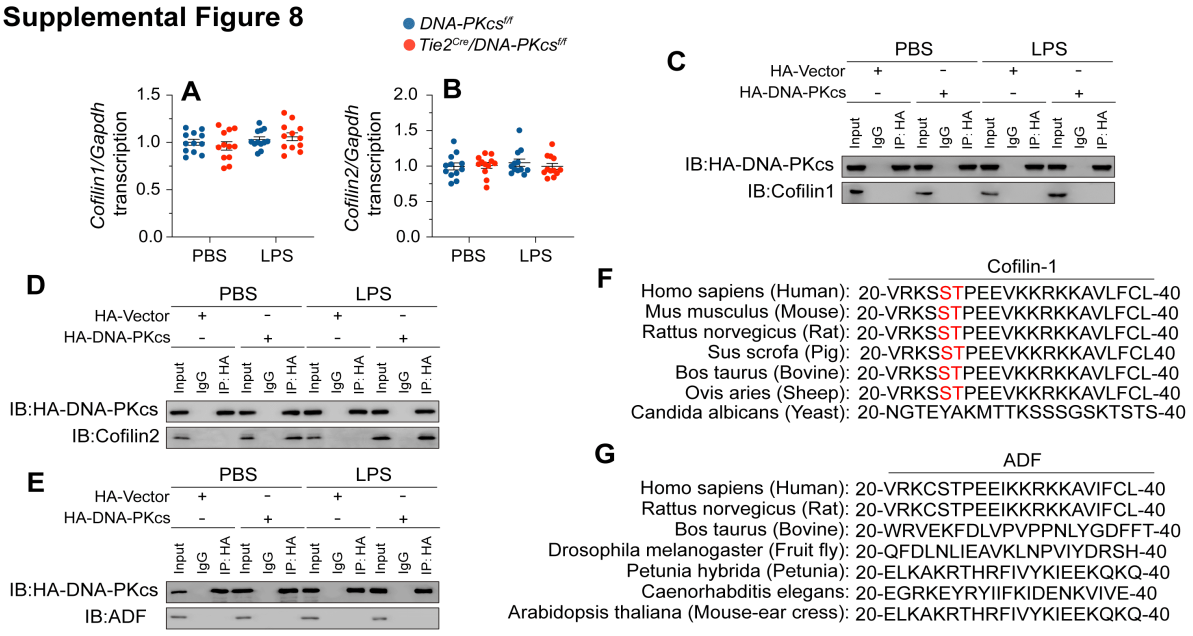

Supplementary Figure 8. DNA-PKcs cannot interact with cofilin1 or ADF. (A, B) RT-qPCR analysis of cofilin1 and cofilin2 expression in CMECs isolated from *DNA-PKcs$^{f/f}$/Tie2^{Cre}* and control *DNA-PKcs$^{f/f}$* mice in the presence of LPS. **(C)** HCAECs were transfected with HA-DNA-PKCs before LPS treatment. Then, the interaction between DNA-PKcs and cofilin1 was measured through Co-IP. **(D)** HCAECs were transfected with HA-DNA-PKCs before LPS treatment. Then, the interaction

between DNA-PKcs and cofilin2 was measured through Co-IP. **(E)** HCAECs were transfected with HA-DNA-PKCs before LPS treatment. Then, the interaction between DNA-PKcs and ADF was measured through Co-IP. **(F)** Amino acid sequences of cofilin1 in various species. **(G)** Amino acid sequences of ADF in various species. Experiments were repeated at least three times. Data are shown as mean ± SEM (n=6 mice or three independent cell isolations per group). *p<0.05.

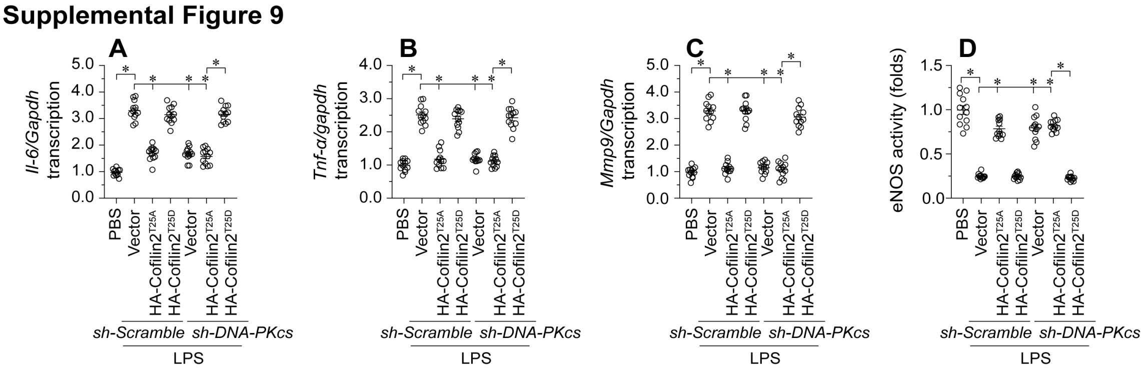

Supplementary Figure 9. Phosphorylation cofilin2 at Thr25 is involved into LPS-mediated endothelial damage. (A-C) RT-qPCR analysis of *Il-6*, *Mmp9* and *Tnf-α* transcription in HCAECs transfected with His-cofilin2^{T25D} and His-cofilin2^{T25A} and exposed to LPS. **(D)** ELISA analysis of eNOS activity in HCAECs transfected with His-cofilin2^{T25D} and His-cofilin2^{T25A} and treated with LPS. Experiments were repeated at least three times and the data are shown as mean ± SEM (n=6 mice per group). Experiments were repeated at least three times. Data are shown as mean ± SEM (n=3 cell isolations per group). *p<0.05.

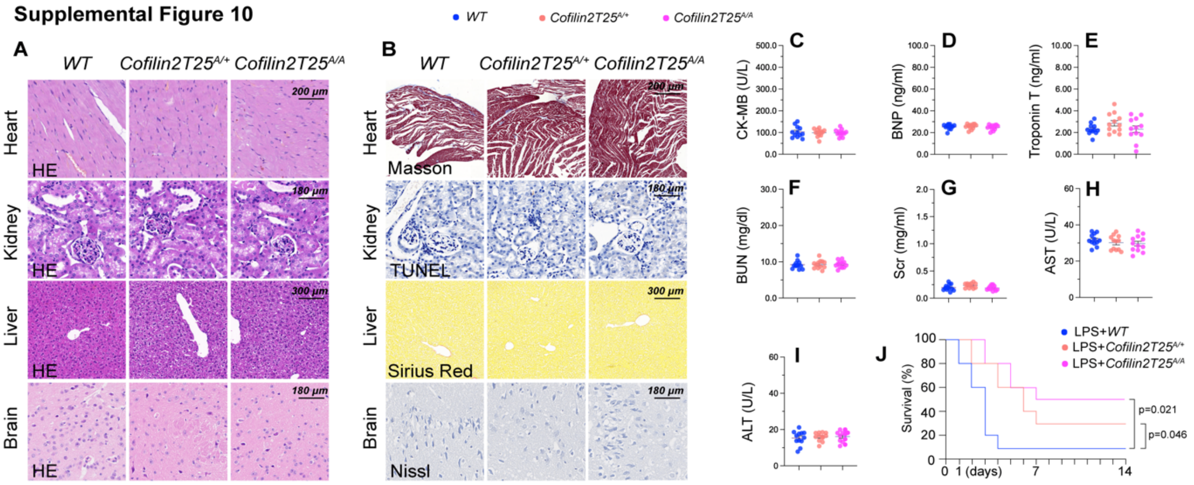

Supplementary Figure 10. The functional and structural alterations of heart, kidney, liver and brain of *cofilin2T25$^{A/+}$* and *cofilin2T25$^{A/A}$* mice. (A) HE staining was used to observe the changes of heart, kidney, liver and brain. **(B)** Pathological staining of various organs. Masson staining was used to observe the cardiac fibrosis of heart. TUNEL staining was used to observe cell death in kidney tissue.

Sirius Red staining was used to detect the hepatic fibrosis in liver. Nissl staining was used to observe neural death in brain. **(C-E)** ELISA was used to analyze the levels of TnT, CK-MB and BNP in the serum of *cofilin2T25*$^{A/+}$ and *cofilin2T25*$^{A/A}$ mice. **(F-G)** BUN and Scr, the indicator of kidney function, were measured using serum isolated from *cofilin2T25*$^{A/+}$ and *cofilin2T25*$^{A/A}$ mice. **(H-I)** AST and ALT, as the markers of hepatic damage, were detected by ELISA using the serum isolated from *cofilin2T25*$^{A/+}$ and *cofilin2T25*$^{A/A}$ mice. **(J)** The survival rate of *cofilin2T25*$^{A/+}$ and *cofilin2T25*$^{A/A}$ mice in the presence of LPS. *$p<0.05$.
